# Genome-wide identification and expression analysis of the MYB transcription factor in moso bamboo (*Phyllostachys edulis*)

**DOI:** 10.7717/peerj.6242

**Published:** 2019-01-11

**Authors:** Kebin Yang, Ying Li, Sining Wang, Xiurong Xu, Huayu Sun, Hansheng Zhao, Xueping Li, Zhimin Gao

**Affiliations:** 1 Institute of Gene Science for Bamboo and Rattan Resources, International Center for Bamboo and Rattan, Beijing, China; 2 State Forestry Administration Key Open Laboratory on the Science and Technology of Bamboo and Rattan, Beijing, China

**Keywords:** *Phyllostachys edulis*, MYB transcription factor, Functional prediction, Gene expression pattern analysis, Secondary cell wall

## Abstract

The MYB family, one of the largest transcription factor (TF) families in the plant kingdom, plays vital roles in cell formation, morphogenesis and signal transduction, as well as responses to biotic and abiotic stresses. However, the underlying function of bamboo MYB TFs remains unclear. To gain insight into the status of these proteins, a total of 85 PeMYBs, which were further divided into 11 subgroups, were identified in moso bamboo (*Phyllostachys edulis*) by using a genome-wide search strategy. Gene structure analysis showed that *PeMYB*s were significantly different, with exon numbers varying from 4 to 13. Phylogenetic analysis indicated that PeMYBs clustered into 27 clades, of which the function of 18 clades has been predicted. In addition, almost all of the *PeMYB*s were differently expressed in leaves, panicles, rhizomes and shoots based on RNA-seq data. Furthermore, qRT-PCR analysis showed that 12 *PeMYB*s related to the biosynthesis and deposition of the secondary cell wall (SCW) were constitutively expressed, and their transcript abundance levels have changed significantly with increasing height of the bamboo shoots, for which the degree of lignification continuously increased. This result indicated that these *PeMYB*s might play fundamental roles in SCW thickening and bamboo shoot lignification. The present comprehensive and systematic study on the members of the MYB family provided a reference and solid foundation for further functional analysis of MYB TFs in moso bamboo.

## Introduction

Secondary cell wall (SCW) deposition and lignification is one of the most important and valuable biological activities for plant growth and development, and the SCW is one of most abundant raw materials on earth and has a wide range of industrial applications (Oh, Park & Han, 2003). Lignified SCWs contribute to the excellent material quality of wood species and lignocellulosic biomass as the most environmentally cost-effective renewable sources of energy (Seth, 2003; Pauly & Keegstra, 2010; Cassan-Wang et al., 2013). In the context of the economic and environmental significance of SCW, the SCWs of plant cells have received increasing attention (Carroll & Somerville, 2009; Pauly & Keegstra, 2010; Cassan-Wang et al., 2013). Great progress had been made in the understanding of the genetic regulation of SCW biosynthesis, which includes several consecutive processes, mainly at the transcriptional level (Hirano et al., 2013). The regulation of SCW biosynthesis involves a complex network that includes transcription factors (TFs) and miRNAs, among which most of the TFs belonging to the MYB family have been reported to function as links between upstream NAC TFs and downstream structural genes (Cassan-Wang et al., 2013; Hussey et al., 2013; Nakano et al., 2015).

The MYB family is one of the largest TF families, and it is named after a highly conservative sequence (MYB DNA-binding domain) located at the N-terminus of these proteins. Each MYB DNA-binding domain comprises 1–4 serial and nonredundant imperfect repeats (R1, R2, R3 and R4). Each repeat contains approximately 50–53 amino acids that form three α-helices, and a helix–turn–helix (HTH) structure is formed between the second and third α-helices (Lipsick, 1996; Stracke, Werber & Weisshaar, 2001). Diametrically, the amino acid sequence outside of the DNA-binding domain, the C-terminus, is an activated structure, that is, highly divergent in length and sequence, which gives rise to the functional diversity of the MYB proteins (Kranz, Scholz & Weisshaar, 2000; Jin & Martin, 1999). Based on the number of repeats, the MYB family is classified into four subfamilies, namely, 1R-MYB (MYB-related), 2R-MYB (R2R3-MYB), 3R-MYB (R1R2R3-MYB) and 4R-MYB (Dubos et al., 2010; He et al., 2016). In plants, R2R3-MYB are the largest and most common MYB TFs (Dubos et al., 2010; Du et al., 2013; Niu, Jiang & Xu, 2016), and these proteins are involved in almost all aspects and stages of plant growth and development. At present, functional studies on MYB have been mainly focused on R2R3-MYB TFs and have rarely addressed the other three TF subfamilies.

To date, many members of the MYB TF family have been recognized and studied in many crops and horticultural plants, such as *Arabidopsis*, poplar, maize, soybean, pineapple, upland cotton and beet (Wilkins et al., 2009; Dubos et al., 2010; Du et al., 2012a, 2012b; Stracke et al., 2014; Salih et al., 2016; Liu et al., 2017). MYB TFs are involved in regulating the growth and development of various plants by participating in many physiological and biochemical processes, such as cell and petal morphogenesis and flavonol biosynthesis (Baumann et al., 2007; Stracke et al., 2007), and one of the most important functions of these proteins is regulating the synthesis of SCWs (Yang & Wang, 2016). Importantly, MYB TFs play central roles in the transcriptional regulation of the deposition of the plant SCW. Many MYB genes have been identified as key genes involved in SCW synthesis, such as *AtMYB58*, *AtMYB63*, *AtMYB46*, *AtMYB83* and *AtMYB103* (Zhou et al., 2009; McCarthy, Zhong & Ye, 2009; Hussey et al., 2011; Guo et al., 2017). In contrast, three other MYB genes, *AtMYB4*, *AtMYB7* and *AtMYB32*, can inhibit the expression of NAC genes, supporting the idea that these genes are negative regulators of SCW synthesis (Zhong et al., 2008). Similarly, homologous genes have also been found in other species. *BplMYB46* of *Betula platyphylla* and *SbMYB60* of *Sorghum bicolor* are involved in the deposition of SCW through regulating lignin synthesis (Zhou et al., 2009; Scully et al., 2016; Guo et al., 2017). However, the function of MYBs related to SCW synthesis in bamboo is still unknown.

Bamboo is characterized by fast growth with a long vegetative period and high yield, which have high value in various industries, such as papermaking, forestry and crafts. Furthermore, young bamboo shoots can be used for food (Wu et al., 2015). Bamboo is also regarded as an emerging and important sources of lignocellulosic biomass energy. The rapid growth of bamboo is accompanied with SCW thickening and lignification, which plays a vital role in the improvement of excellent wood property for broad application in the manufacturing industry (Yu, 2003; Gao et al., 2010). MYB TFs are involved in SCW development by regulating the expression of lignin, cellulose and hemicellulose synthesis-related genes and therefore indirectly affect material properties (Hussey et al., 2013; Soler et al., 2015). In addition, the number of MYB TF family members greatly varies from species to species, and even homologous genes differ in gene structure and function among different species. Therefore, it is necessary to explore the specific structural and functional characteristics of bamboo MYB genes for further study. The goal of this study is to fully understand the status of MYB TFs related to secondary wall synthesis in moso bamboo.

Moso bamboo (*Phyllostachys edulis*) is an important woody bamboo with high value for lignocellulosic biomass and is the only bamboo that has been sequenced. In moso bamboo, we hypothesize that (i) the members of the MYB TF family might have similar gene structures and different numbers of family members compared to those in *Arabidopsis thaliana*; (ii) the expression pattern of MYB genes may show significant tissue specificity; (iii) and the MYB genes would primarily be involved in the biosynthesis and deposition of the SCW. To test these hypotheses, we identified MYB genes in the whole genome of moso bamboo and investigated their gene structural characteristics and their evolutionary relationships. We further investigated the tissue-specific expression patterns of these genes by using RNA-seq data. Finally, we validated the role of MYB genes related to the biosynthesis of SCW by qRT-PCR using bamboo shoots with different lignification degrees. Thus, the present study provided a starting point for further functional analysis of MYB genes in moso bamboo, and laid the foundation of selecting candidate genes for genetic engineering in bamboo breeding.

## Materials and Methods

### Plant material

To examine expression differences of the MYB genes involved in the biosynthesis of SCWs during the lignification of shoots in moso bamboo, the basal parts of bamboo shoots with different heights (0.2, 1.0, 3.0 and 6.7 m) were collected from the bamboo forest experimental site of Jiangxi Academy of Forestry located in Nanchang City, Jiangxi Province, China (E115°46′1″; N28°45′57″). Shoots of 0.2, 1.0, 3.0 and 6.7 m in height correspondingly belonged to preliminary, ascending, prosperous and late shoot developmental stages, in which the degree of lignification gradually increased. These mixed samples from three individual shoots with different heights were immediately frozen in liquid nitrogen and stored at −80 °C until further use. Meanwhile, a part of the same samples had been fixed in formalin-acetic acid-alcohol (FAA) and kept in a refrigerator at 4 °C before use.

### Histological methods

Shoot samples were taken out from FAA fixative, dehydrated through a graded series of polyethylene glycol (PEG) (Tianjin Guangfu Fine Chemical Research Institute, Tianjin, China) at 80 °C, first with the equal volume mixture of PEG 1000 and deionized water, followed by the equal volume mixture of PEG 1000 and PEG 4000, and finally with PEG 4000. The samples embedded in PEG 4000 at room temperature were used for section making with a rotary microtome (Leica RM2165; Leica, Frankfurt, Germany). Tissue sections (10 μm) were cut transversely from the embedded samples and gently transferred to clean slides with brushes. The sections were fully expanded and stained with toluidine blue (Windham et al., 2018), which were heated at 80 °C for 3 min. The slides were washed with deionized water and dried. Cover slips were cemented over stained sections and viewed with an Olympus CX31 microscope.

### Database search for MYBs in moso bamboo

The genome of moso bamboo (Peng et al., 2013b) and the BambooGDB database (http://www.bamboogdb.org/) (Zhao et al., 2014) facilitated a genome-wide analysis of the bamboo gene families (Sun et al., 2016; Huang et al., 2016). To identify the potential members of the MYB TF family in moso bamboo, we performed multiple sequence blast and alignment. First, the putative MYB sequences were downloaded from BambooGDB. Then, the MYB conserved domains in putative sequences were examined by using BLASTN and BLASTP. Finally, all MYB sequences were manually inspected to ensure that the putative protein models contained two, three and four MYB repeats, and the protein sequences that did not contain conserved domains were deleted. The MYB TFs were named according to their BambooGDB assembly names.

The basic characteristics of the potential MYB TF members in moso bamboo were further analyzed, including the predicted proteins and the physicochemical parameters. The predicted molecular weights (MWs) and isoelectric points (pIs) of the MYB proteins were analyzed using ProtParam (http://web.expasy.org/protparam/).

### WebLogo and gene structure analysis

To reveal the sequence features of the conserved DNA-binding domains in R2R3-MYB proteins, the sequences of the R2 and R3 MYB repeats in all PeR2R3-MYB proteins were compared using the ClustalW program in MEGA (version 6.0) (Tamura et al., 2013). The same method was used to perform the multiple sequence alignment encompassing 82 R2R3-MYB proteins from moso bamboo, 126 from *A. thaliana* and 111 from *Oryza sativa* (rice). The distribution of the amino acid residues at the corresponding positions in the conserved domains of R2R3-PeMYBs were generated using the WebLogo program with default parameters (http://weblogo.berkeley.edu/logo.cgi) (Crooks et al., 2004).

For the gene structure analysis, the exons and introns of the MYB genes (*PeMYB*s) were illustrated using the Gene Structure Display Server (GSDS; http://gsds.cbi.pku.edu.cn/) (Guo et al., 2007) to align the cDNA sequences with the corresponding genomic DNA sequences from the BambooGDB.

### Phylogenetic analysis and function prediction

To explore the evolutionary relationships among MYBs in moso bamboo and predict the functions of these MYBs, the MYB sequences of *Arabidopsis* and rice were downloaded from the *Arabidopsis* genome TAIR (The *Arabidopsis* Information Resource) release 10.0 (http://www.arabidopsis.org/) and the rice genome annotation database (http://rice.plantbiology.msu.edu/index.shtml, release 7.0). A neighbor-joining (NJ) phylogenetic tree was constructed with ClustalW to align the full-length of MYB amino acid sequences (85 PeMYBs and 132 AtMYBs) using MEGA (version 6.0) with the following parameters: Poisson correction, pairwise deletion, and bootstrap analysis with 1,000 replicates. The PeMYBs were classified according to their phylogenetic relationships with the corresponding 27 clades of AtMYBs (Stracke, Werber & Weisshaar, 2001; Dubos et al., 2010). Additionally, the biological functions of PeMYBs were predicted according to the aforementioned phylogenetic tree and previously studies homologous *Arabidopsis* proteins with validated specific function (Zhong et al., 2008; McCarthy, Zhong & Ye, 2009; Dubos et al., 2010; Li et al., 2016b).

### Tissue specific expression analysis of MYB genes

To study the expression patterns of MYB genes in different tissues of moso bamboo, the transcriptome data for the leaves, panicles, roots, rhizomes and shoots at different developmental stages were downloaded from the Short Read Archive of NCBI. The gene expression abundance was calculated by the reads per kilobase per million mapped reads value of each MYB gene. For the convenience of statistics, logarithm (Log) was used for each expression as base 2. The heat map of gene expression was decorated using Matrix2png (http://www.chibi.ubc.ca/matrix2png/).

### Real-time PCR analysis

Total RNA was extracted using the plant RNA extraction kit (Qiagen, Dusseldorf, Germany) according to the manufacturer’s instructions. The integrity of the total RNA was verified through agarose gel electrophoresis, and the purity and concentration of the total RNA was determined by spectrophotometry. The first strand cDNA was synthesized by a reverse transcription system (Promega, Madison, WI, USA). For each 20-μL reaction, 1,000 ng of total RNA was used, and the synthesis was performed at the 42 °C for 45 min. The final cDNA product was diluted fivefold prior to use.

*PeMYB*s involved in SCW synthesis were screened according to their phylogenetic relationships with the corresponding *AtMYB*s. Based on the multiple alignments, 12 specific primers for different *PeMYB*s were designed by Primer Premier 5.0 software and empirically adjusted for gene expression analysis (Table S1). Additionally, all primers showing a clear specific melting peak by real-time melting curve analysis, consistent with the results of agarose gel electrophoresis for specific PCR products, were used for further analysis. The qRT-PCR was performed with the Roche Light Cycler 480 SYBR Green 1 Master kit on a qTOWER2.2 system (Analytik Jena, Jena, Germany). The qRT-PCR program involved 95 °C for 10 min, followed by 40 cycles at 95 °C for 10 s and 60 °C for 10 s. The 10.0 μL reaction volume contained 5.0 μL of 2× SYBR Green 1 Master Mix, 0.8 μL of cDNA, 0.1 μL of primer (10.0 mM, each) and 4.0 μL of ddH_2_O. *NTB* and *TIP41* were used as reference genes (Fan et al., 2013). The 2^−ΔΔCt^ method was used for the analysis and visualization of real-time PCR data generated by multiple technical replicates (Liu et al., 2013).

### Statistical analysis

Analyses were performed with SPSS Statistics for Windows (Version 22.0; SPSS Inc., Chicago, IL, USA). All data were the average and standard error of three biological replicates. One-way analysis of variance was used to evaluate the statistical significance of differences among means using SPSS software. Single and double asterisks indicate significant differences at the levels of *p* < 0.05 and *p* < 0.01, respectively.

## Results

### Identification, protein characteristics and conserved DNA-binding domain analysis of MYB TFs in moso bamboo

Through comprehensive comparison analysis, we identified a set of 85 MYB proteins containing MYB DNA-binding domains in moso bamboo (Table S2), which included 82 typical R2R3-MYB proteins (2R-MYB), two R1R2R3-MYB proteins (3R-MYB) and one 4R-like MYB protein (4R-MYB). According to the numbering order of MYB in BambooGDB, 2R-MYB proteins were named PeMYB1–PeMYB82, while 3R-MYB and 4R-MYB proteins were named PeMYB3R-1–PeMYB3R-2 and PeMYB4R-1, respectively. As shown in Table 1, the length of the corresponding estimated polypeptides ranged from 199 to 1,024 amino acids, the calculated MW of PeMYBs ranged from 22.3 to 112.4 kDa, and the calculated theoretical pI of PeMYBs was from 5.15 to 11.67. The majority of R2R3-MYB proteins were approximately 300 amino acids with MWs of approximately 30 kDa. However, 82 R2R3-MYB proteins presented irregular characteristics of theoretical pI, leading to 50 acid proteins and 32 basic proteins. Among the 85 PeMYBs, PeMYB3R-2 was the longest protein with 1,024 amino acids, while the shortest protein was PeMYB49 with 199 amino acids.

**Table 1:** Nomenclature and protein information of MYBs in moso bamboo.

Nomenclature used for this paper	Bamboo GDB assembly name	Pl (aa)	MW (Da)	pI	Nomenclature used for this paper	Bamboo GDB assembly name	Pl (aa)	MW (Da)	pI
PeMYB1	PH01000001G2130	277	30,332.85	7.08	PeMYB44	PH01000847G0490	275	30,661.58	5.38
PeMYB2	PH01000005G1390	286	31,377.37	6.24	PeMYB45	PH01000912G0430	247	27,623.01	5.68
PeMYB3	PH01000006G2680	378	40,189.57	5.94	PeMYB46	PH01000958G0180	988	111,211.3	5.15
PeMYB4	PH01000008G0500	288	30,732.33	7.02	PeMYB47	PH01001022G0490	254	27,279.34	8.30
PeMYB5	PH01000008G3080	308	34,411.17	5.43	PeMYB48	PH01001064G0370	392	43,742.65	5.75
PeMYB6	PH01000009G0060	554	59,864.36	4.95	PeMYB49	PH01001084G0440	199	22,335.16	9.78
PeMYB7	PH01000014G1850	257	27,923.20	7.54	PeMYB50	PH01001133G0430	228	26,192.18	8.83
PeMYB8	PH01000028G0940	284	30,699.39	6.18	PeMYB51	PH01001174G0490	441	49,726.99	6.06
PeMYB9	PH01000029G1950	587	63,493.82	6.17	PeMYB52	PH01001208G0070	204	23,122.78	6.31
PeMYB10	PH01000030G0050	363	39,417.23	5.03	PeMYB53	PH01001287G0090	253	28,133.68	6.77
PeMYB11	PH01000041G2150	332	35,798.18	6.87	PeMYB54	PH01001342G0270	272	30,538.57	10.6
PeMYB12	PH01000043G2100	232	25,140.23	9.01	PeMYB55	PH01001430G0250	262	28,784.47	7.60
PeMYB13	PH01000053G1340	309	34,152.74	6.55	PeMYB56	PH01001622G0290	318	33,660.00	9.13
PeMYB14	PH01000060G0800	276	30,672.17	5.90	PeMYB57	PH01001925G0330	247	27,606.01	7.72
PeMYB15	PH01000064G1730	300	32,262.85	7.01	PeMYB58	PH01001991G0310	316	36,090.86	8.52
PeMYB16	PH01000066G1200	273	29,409.31	9.26	PeMYB59	PH01002000G0040	235	25,734.12	8.93
PeMYB17	PH01000068G1000	337	36,928.09	8.46	PeMYB60	PH01002082G0250	284	30,453.86	6.40
PeMYB18	PH01000068G1470	308	32,931.66	6.96	PeMYB61	PH01002092G0300	386	42,365.66	5.04
PeMYB19	PH01000177G0890	304	32,461.31	9.46	PeMYB62	PH01002104G0150	278	30,138.61	9.81
PeMYB20	PH01000198G1320	313	34,110.22	7.66	PeMYB63	PH01002139G0430	289	32,202.79	8.33
PeMYB21	PH01000209G0490	316	34,250.01	7.84	PeMYB64	PH01002184G0220	325	34,980.08	4.63
PeMYB22	PH01000212G0840	424	46,705.17	6.32	PeMYB65	PH01002276G0160	328	35,201.40	6.87
PeMYB23	PH01000234G0090	328	35,312.59	6.68	PeMYB66	PH01002680G0080	273	30,149.20	5.81
PeMYB24	PH01000302G0910	295	32,877.25	5.76	PeMYB67	PH01002704G0220	313	35,029.41	6.38
PeMYB25	PH01000305G0990	263	29,168.77	9.74	PeMYB68	PH01002707G0220	304	33,482.32	6.10
PeMYB26	PH01000332G0140	240	27,343.46	8.85	PeMYB69	PH01002736G0020	426	47,126.77	6.17
PeMYB27	PH01000345G0740	266	29,911.06	5.90	PeMYB70	PH01002868G0200	272	29,620.53	9.41
PeMYB28	PH01000374G0780	509	56,256.12	8.06	PeMYB71	PH01003180G0140	327	35,295.85	6.06
PeMYB29	PH01000386G0660	346	37,877.37	5.23	PeMYB72	PH01003507G0040	294	31,738.31	5.44
PeMYB30	PH01000392G0510	339	36,821.16	5.63	PeMYB73	PH01003809G0130	341	37,143.94	5.15
PeMYB31	PH01000415G0010	292	31,593.87	7.68	PeMYB74	PH01003918G0100	445	49,270.93	5.90
PeMYB32	PH01000415G0090	284	30,871.39	6.31	PeMYB75	PH01004818G0120	272	30,731.28	6.40
PeMYB33	PH01000427G0040	261	28,722.34	7.03	PeMYB76	PH01004865G0070	240	27,157.40	11.67
PeMYB34	PH01000445G0700	310	34,607.94	5.64	PeMYB77	PH01005192G0010	289	30,825.37	6.35
PeMYB35	PH01000462G0290	322	35,252.55	5.59	PeMYB78	PH01005460G0120	297	32,145.28	8.11
PeMYB36	PH01000501G0490	321	34,627.14	7.69	PeMYB79	PH01005515G0070	357	39,436.22	6.51
PeMYB37	PH01000508G0100	334	36,499.74	5.85	PeMYB80	PH01005685G0080	338	36,493.24	6.06
PeMYB38	PH01000515G0560	276	31,171.97	5.61	PeMYB81	PH01005828G0060	317	35,395.00	5.40
PeMYB39	PH01000569G0800	264	28,563.96	5.50	PeMYB82	PH01007341G0010	272	29,204.97	9.20
PeMYB40	PH01000595G0350	427	47,009.56	6.23	PeMYB3R-1	PH01000054G0640	817	90,393.02	9.36
PeMYB41	PH01000604G0860	236	26,573.87	7.71	PeMYB3R-2	PH01000812G0550	1,024	112,448.60	5.22
PeMYB42	PH01000617G0820	275	30,759.97	5.50	PeMYB4R-1	PH01002148G0200	930	102,980.90	9.23
PeMYB43	PH01000729G0470	218	23,255.01	8.25					

**Note:**

Pl, polypeptide length; MW, molecular weight; Da, Dalton; pI, isoelectric point; aa, amino acids.

**Figure 1:**
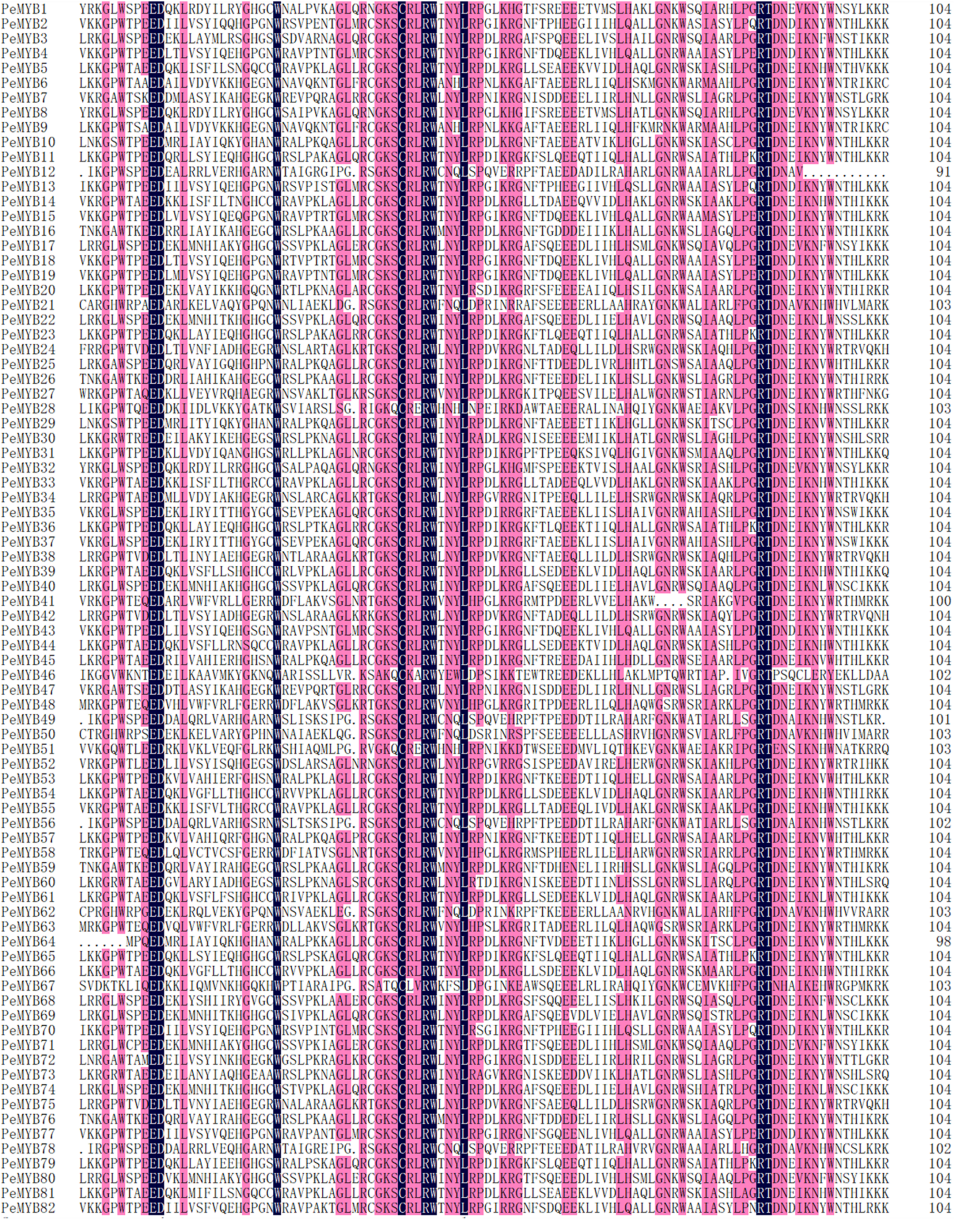
Multiple alignment of the amino acid sequences of 82 moso bamboo R2R3-MYB domains.

To further investigate and identify the characteristics of homologous domains in R2R3-MYB proteins, multiple sequence alignment and WebLogo were performed using the amino acid sequences of R2 and R3 repeats in 82 PeMYBs (R2R3-MYB). As shown in Fig. 1 and Fig. S1, R2R3-MYB proteins contained R2 and R3 repeats, suggesting that the characterized PeMYBs were similar to those of other species, with basic R2 and R3 structures of [-W-(X19)-W-(X19)-W-] and [-F-(X18)-W-(X18)-W-], respectively. The results showed that each repeat included a highly conserved triplet of tryptophan (W) residues, and each characteristic of W was separated by 18 or 19 amino acids, which were located at positions 6, 26 and 46 of the R2 repeat and 58, 77 and 96 of the R3 repeat (Fig. 1; Fig. S1). In addition to the highly conserved W, glutamic (E)-10, aspartic (D)-11 cysteine (C)-42, arginine (R)-45 in the R2 repeat, leucine (L)-50 in the linker region, and arginine (R)-87, threonine (T)-88 in the R3 repeat were also completely conserved (Fig. 1; Fig. S2). Interestingly, the first conservative tryptophan (located at 58) in R3 was mostly substituted by phenylalanine (F), marginally by isoleucine (I) and leucine (L), and the individual tryptophan was conserved. Furthermore, the glycine (G) (located at 74) was replaced by alanine (A) for PeMYB73 and PeMYB30, and threonine (T) for PeMYB60, respectively. As shown in Fig. 1 and Fig. S1, the conservative areas in the MYB DNA-binding domain were mainly located between the second and third W of the two R repeats (the third helix of the HTH domain in each repeat). However, the amino acid sequence between the first and second W of each R repeat in the MYB DNA-binding domain was relatively unconserved.

### Phylogenetic analysis of MYB TFs based on gene structure

**Figure 2:**
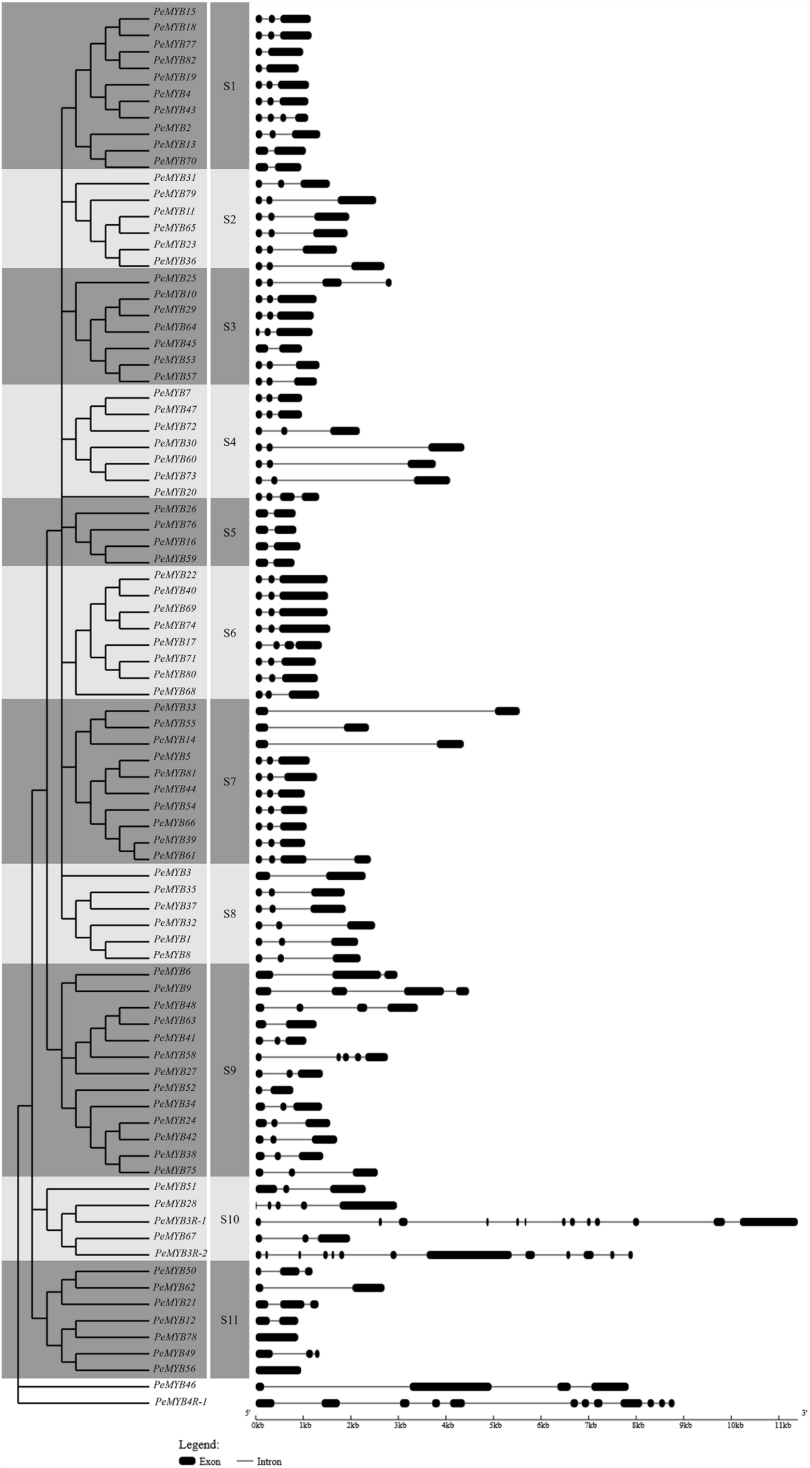
Phylogenetic relationships and gene structures of MYB genes in moso bamboo. The amino acid sequences of 85 PeMYBs were aligned by the Clustal W program in MEGA, and the phylogenetic tree was constructed by the NJ method with 1,000 bootstrap replicates. Bootstrap values >50 were indicated on the nodes. Different subgroups were marked with alternating tones of a gray background to make subgroups identification easier. Exon/intron structures of the *PeMYB*s: black boxes represented exons and spaces between the black boxes correspond to introns.

There was a significant difference and diversity in the gene structure among the members of *PeMYB*s, including the number and relative location of exons and introns. As shown in Fig. 2, *PeMYB56* and *PeMYB78* had no introns, while the number of introns in the other members varied from 1 to 13. According to their predicted structures, the majority of *PeMYB*s contained two or three exons, and the number of members with the above characteristics was 17 and 53, respectively. To reveal the phylogenetic relationship of the MYB proteins, we performed multiple sequence alignment using the amino acid sequences of 85 PeMYBs. According to the similarity and systematic evolution of the sequences, PeMYBs were divided into 11 subgroups (designated as S1–S11) and two low homology MYB proteins (PeMYB46 and PeMYB4R-1), and each subgroup had 4–13 members. The most highly homologous members in the same subgroup generally shared the same or parallel exon/intron patterns, showing similar quantity, location and exon length. For instance, four *PeMYB*s (*PeMYB26*, *PeMYB76*, *PeMYB16* and *PeMYB59*) in S5 included two exons and one intron. Notably, one or more pairs of PeMYBs with highly homology were found in the terminal nodes of each subgroup, suggesting that these proteins share similar functions. There was also an exception of S9, in which the location and length of exons and introns were significantly different with low genetic similarity among the members.

### Putative functions of PeMYBs in moso bamboo

**Figure 3:**
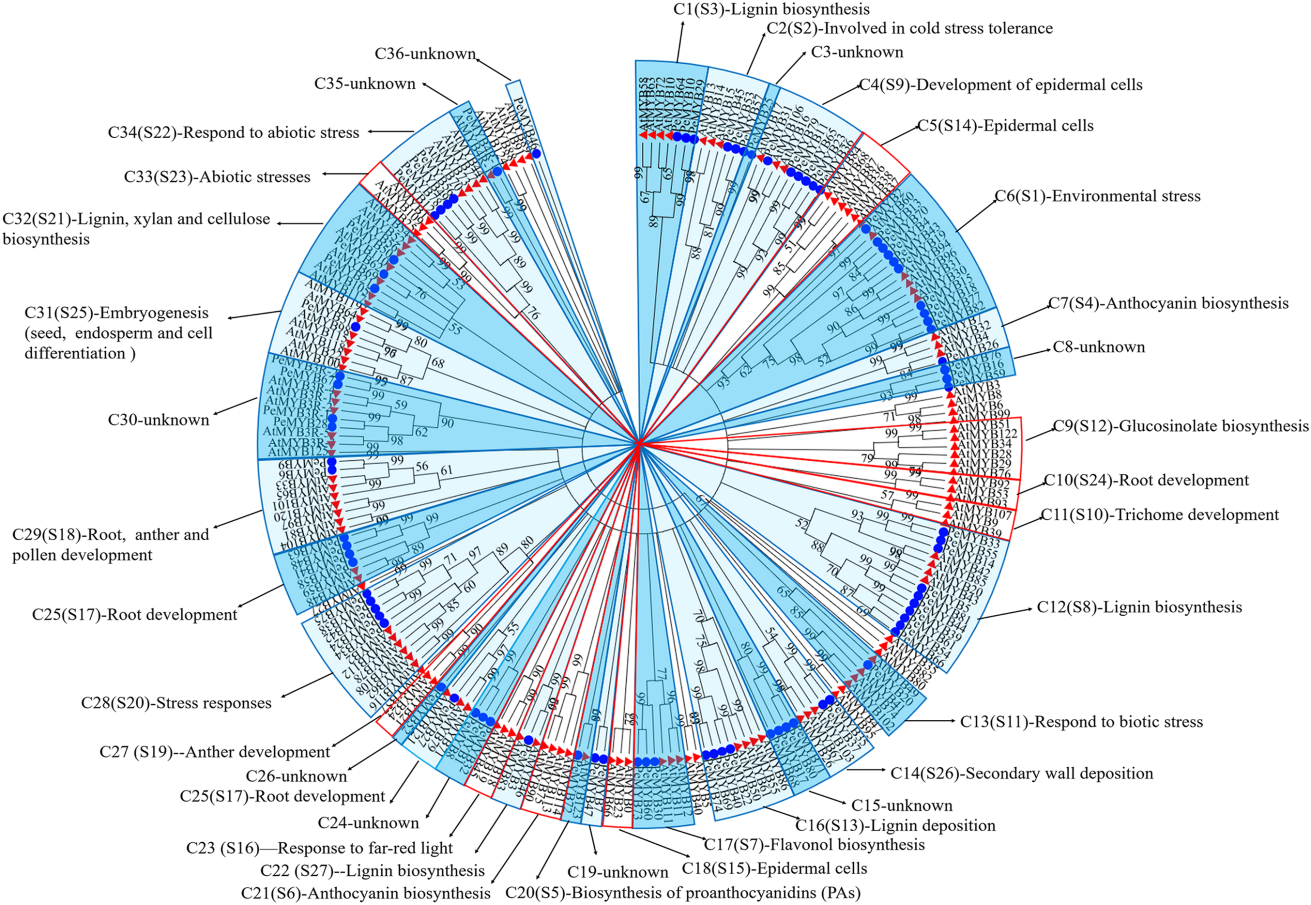
Putative functions of the MYB proteins in moso bamboo based on the phylogenetic tree along with MYBs from Arabidopsis. The circular unrooted tree was generated by NJ method with 1,000 bootstrap replicates. Different subclades were marked with alternating tones of a blue background. The red boxes indicated that species-specific subclades of Arabidopsis.

MYB TFs in *A. thaliana* are divided into 27 clades, and the function of each clade had been annotated (Zhong et al., 2008; McCarthy, Zhong & Ye, 2009; Dubos et al., 2010; Li et al., 2016b). It is assumed that homologous proteins that clustered together typically have similar functions, suggesting that the PeMYBs had similar functions as AtMYBs in the same clade. Therefore, the functions of PeMYBs were predicted and summarized by comparison with those of AtMYBs (Fig. 3; Table S3). An NJ unrooted phylogenetic tree was constructed using 85 PeMYBs and 132 AtMYBs (Fig. 3). The results showed that all MYB members from the two species were clustered into 36 clades (designated as C1–C36), including 20 clades common to the two species, and seven and nine species-specific clades of moso bamboo and *Arabidopsis*, respectively.

According to the above analysis, 65 PeMYBs belonging to 18 function-annotated clades, and 20 PeMYBs belonging to nine function-unknown clades were found. Based on the annotation, 65 PeMYBs were divided into four functional classes. Class I, including six clades (C1, C12, C14, C16, C22 and C32), was responsible for SCW formation by regulating the biosynthesis and deposition of lignin, cellulose and hemicellulose. Class II, including five clades (C2, C6, C13, C28 and C34), was involved in responses to biotic and abiotic stresses by regulating the ABA pathway. Class III, including 5 clades (C4, C20, C25, C29 and C31), played important roles in morphogenesis and organogenesis, such as root, epidermal cell, anther, vegetative and stomatal cell development and embryogenesis. Class IV, including 2 clades (C7 and C17), was involved in regulating secondary metabolism, such as anthocyanins and flavonols biosynthesis. Class IV, including two clades (C4 and C31), was involved in regulating secondary metabolism, such as anthocyanins and flavonols biosynthesis. Thus, these results suggested that PeMYBs have a wide range of functions and may play important roles in the growth and development of moso bamboo.

### Tissue-specific expression analysis of *PeMYB*s by using the transcriptome data of moso bamboo

**Figure 4:**
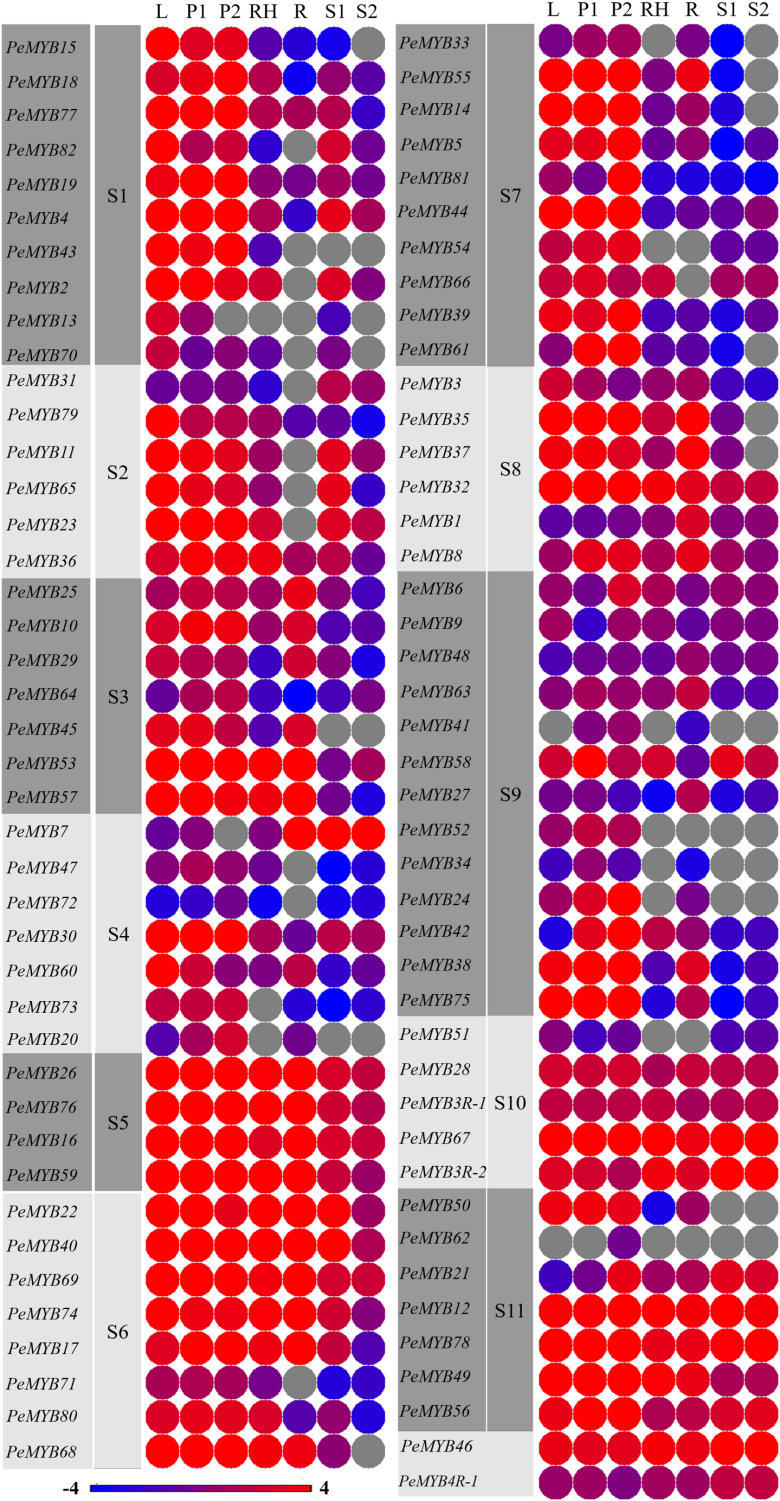
Expression profiles of *PeMYB*s in different tissues and development stages. Heatmap showing the expression of 85 *PeMYB*s in different tissues analyzed. Color scale at the bottom of the picture represents log_2_ expression values: blue indicating low level and red indicating high level of transcript abundance. L, leaves; P1, early panicles; P2, advanced panicles; R, roots; Rh, rhizomes; SH1, 0.2 m shoots; SH2, 0.5 m shoots.

The tissue-specific expression of *PeMYB*s was analyzed by constructing a heat map using the transcriptome data of moso bamboo (Peng et al., 2013b). The results showed significant differences in the expression profiles of *PeMYB*s in different tissues, and most of the *PeMYB*s showed significant tissue specificity. The expression of all 85 *PeMYB*s was detected in at least one tissue, and 52 *PeMYB*s were expressed in all tissues, with transcript abundances varying from 0 to 110.12 (Fig. 4). Furthermore, some *PeMYB*s showed high expression in a particular tissue, for example, *PeMYB*s belonging to S1, S2 and S7 mostly showed dominant expression patterns in the leaves and panicles, and relatively low expression in the other four tissues. The detailed analysis of the expression patterns of *PeMYB*s showed that eight *PeMYB*s (*PeMYB12*, *PeMYB16*, *PeMYB26*, *PeMYB32*, *PeMYB46*, *PeMYB67*, *PeMYB69* and *PeMYB78*) showed dominant expression in almost all tested tissues. Interestingly, the expression of each gene in shoots was relatively lower, and nine *PeMYB*s were not detected in bamboo shoots (Fig. 4). In addition, *PeMYB*s belonging to S5 and S6 showed high expression in all tissues, whereas *PeMYB*s belonging to S9 showed low expression in all tissues. Moreover, *PeMYB62* was exclusively expressed in advanced panicle samples.

### Validation of *PeMYB*s by using real-time PCR

The function prediction results indicated that many *PeMYB*s belonged to clades related to the biosynthesis and deposition of SCW. Thus, 12 *PeMYB*s, including *PeMYB10*, *PeMYB29* and *PeMYB22* in C1, *PeMYB26* in C7, *PeMYB14* and *PeMYB33* in C12, *PeMYB37* in C14, *PeMYB22*, *PeMYB40* and *PeMYB74* in C16, *PeMYB3* in C22 and *PeMYB50* in C32, were selected for further validation. The expression profiles of the selected *PeMYB*s in moso bamboo shoots of different heights were examined by using qRT-PCR with *PeTIP41* as the reference gene.

**Figure 5:**
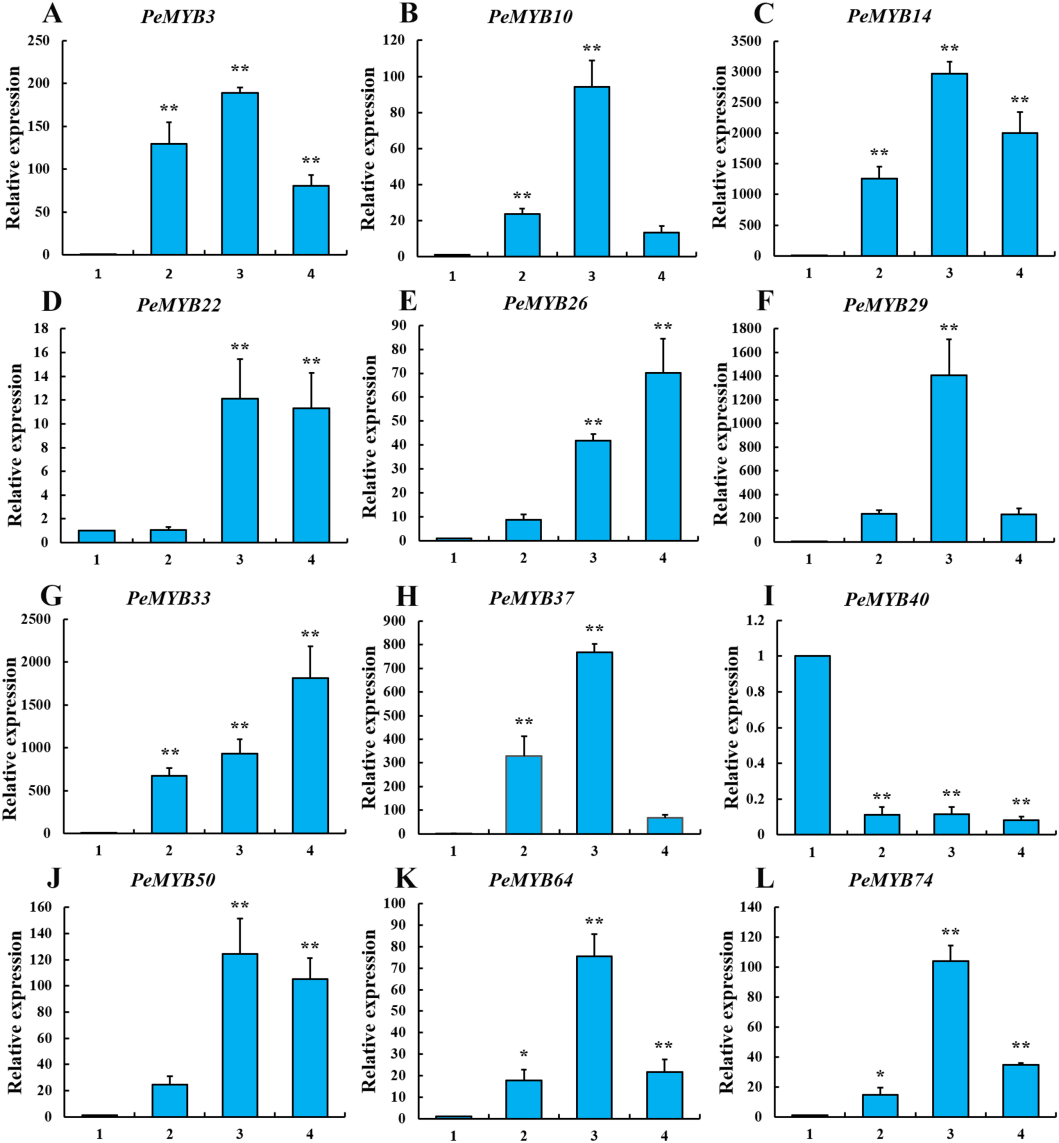
Expression analysis of 12 *PeMYB*s using qRT-PCR. *PeTIP41* was used as the reference gene. Average and error bars represent standard deviation of three biological replicates. Asterisks indicate a significant difference between the higher shoots and the 0.2 m shoots (*p < 0.05, **p < 0.01). (A) *PeMYB3*, (B) *PeMYB10*, (C) *PeMYB14*, (D) *PeMYB22*, (E) *PeMYB26*, (F) *PeMYB29*, (G) *PeMYB33*, (H) *PeMYB37*, (I) *PeMYB40*, (J) *PeMYB50*, (K) *PeMYB64* and (L) *PeMYB74*. 1: 0.2 m shoots; 2: 1.0 m shoots; 3: 3.0 m shoots; 4: 6.7 m shoots.

The results showed that all 12 *PeMYB*s have changed significantly with three expression patterns: a continuously increasing trend, an increasing trend with a final decrease and a trend of slightly stable after a sharp drop with increasing bamboo shoot height. As shown in Figs. 5E and 5G, two *PeMYB*s (*PeMYB26* and *PeMYB33*) were significantly upregulated, and their relative expression were upregulated more than 70 times in 6.7 m shoots compared with that in 0.2 m shoots, particularly the expression of *PeMYB33* was the most significant at 1,810 times. In addition, the expression of *PeMYB40* was found specially, which had a totally different trend with a sharp drop in 1.0 m shoots compared to that in 0.2 m shoots, and then kept at a relatively stable low level.

However, nine *PeMYB*s (*PeMYB3*, *PeMYB10*, *PeMYB14*, *PeMYB22*, *PeMYB29*, *PeMYB37*, *PeMYB50*, *PeMYB64* and *PeMYB74*) exhibited a similar increasing expression trend, which first showed a gradual increase with a peak in 3.0 m shoots, and then a decrease in 6.7 m shoots (Figs. 5A–5D, 5F and 5H–5L). Except *PeMYB3*, all the other eight *PeMYB*s were significantly upregulated more than two times in 3.0 m shoots compared with those in 1.0 m shoots, and the highest one was *PeMYB74* with more than seven times. The relative expression levels of *PeMYB3*, *PeMYB14*, *PeMYB29*, *PeMYB37*, *PeMYB50* and *PeMYB74* in 3.0 m shoots were upregulated more than 100 times of those in 0.2 m shoots, especially for that of *PeMYB14* and *PeMYB29* up to 2,976 and 1,407 times, respectively. Interestingly, there showed three expression decline patterns in 6.7 m shoots compared to that in the 3.0 m shoots, that is, the expression of *PeMYB10*, *PeMYB29*, *PeMYB37* and *PeMYB64* showed an extremely significant decrease of more than 70%, while that of *PeMYB3* and *PeMYB74* was roughly reduced by half and the expression of *PeMYB22* and *PeMYB50* decreased by approximately 10%.

In general, the lignification degree increased with the growth of bamboo shoots, which was confirmed by the histological slides of different height shoots (Fig. S3). Thus, the higher the bamboo shoots, the higher the degree of lignification. These qRT-PCR results indicated that *PeMYB*s are differentially expressed in bamboo shoots of different height, suggesting that these proteins play different important roles in the lignification of the SCW in moso bamboo. Moreover, the expression of all the *PeMYB*s was verified by using *PeNTB* as a second reference gene, which showed similar results (Fig. S4) and strengthened the reliability of the data generated by using *TIP41*.

## Discussion

The MYB family, as one of the largest and most significant TF families in plants, is involved in regulating various process of plant growth and development and most importantly, the transcriptional regulation of SCW deposition (Yang & Wang, 2016; Wei et al., 2017; Sun et al., 2017). MYB TFs play a vital role in the improvement of material properties and the accumulation of lignocellulosic biomass (Zhong, Richardson & Ye, 2007; Zhong et al., 2008; Zhou et al., 2009). However, little is known about the MYB TFs in bamboo, and less is known about their specific functions in the formation and deposition of SCW. Therefore, the present study focused on the analyses of the gene structure, evolutionary relationship, tissue-specific expression and function prediction of *PeMYB*s, which provided the basis for the further study and practical application of *PeMYB*s.

### Homology of gene structure indicated close evolutionary relationships and similar functions

In the present study, 85 PeMYBs were identified in moso bamboo, and the number of PeMYB members was slightly less than that found in other monocotyledonous and dicotyledonous plants (Dubos et al., 2010; Li et al., 2016c; Zhang et al., 2016). It is likely that the bamboo genome is a draft, which does not cover the entire genome (Peng et al., 2013a). In the present study, the members of the MYB gene family were divided into the same subgroups with mostly similar exon/intron structures (Fig. 2). Interestingly, the exons of most of the members in a same subgroup were relatively conserved. For example, the first or first two exons had the same size and position but showed differences in intron length and the position of the last exon, which resulted in a shift in the intron splicing position. This finding suggested that these members were evolutionarily close and might share a similar function. Similarly, previous studies suggested that homologous MYB proteins that cluster together in a subgroup or clade share similar or the same evolutionary origins, which is particularly reflected in the gene structure of the number and size of exons and introns as well as the insertion position of introns (Dubos et al., 2010; Li et al., 2016c). However, there were also some exceptions with different gene structures from other *PeMYB*s in the same subgroup, which indicated that there might be differences in the evolution of the members in the MYB family and these proteins might have new functions. This finding further supported the diversity of MYB function.

### The variation of amino acids in the conserved DNA-binding domain might change the function and activity

In general, MYB proteins possessed the characteristic of a highly conserved DNA-binding domain in the N-terminus, and the second half of each R structure was particularly conserved (Kranz, Scholz & Weisshaar, 2000; Jiang, Gu & Peterson, 2004; Dubos et al., 2010; Du et al., 2012b). In the present study, the third helical structures of PeMYBs were more conserved than the other two helical structures, consistent with the findings of previous studies (Jiang, Gu & Peterson, 2004; Du et al., 2012b). We speculated that the amino acid sequence of the third helix was particularly important for the function of PeMYBs. On the one hand, the highly conserved amino acids in the third helix may reflect the functional stability of MYBs throughout the long evolutionary process in plant. Moreover, further analysis of a typical characteristic structure from other model pants (*Arabidopsis* and rice) indicated that threonine (T)-88 in the R3 repeats were completely conserved in 82 PeR2R3-MYBs, while the change from threonine (T)-88 to S or tyrosine (Y) was observed in three AtMYBs and seven OsMYBs. This reflected the functional divergence among different plant species (Fig. 1; Figs. S1 and S2).

On the other hand, the species-specific function of PeMYBs may result from the variation of the key amino acids in this region. In addition, the replacement of amino acids within the linker region between R2 and R3 repeats was found in PeMYBs. For instance, the proline (P)-52 in the linker region was substituted by serine (S), which may decrease the stability of the protein-DNA complex and even lead to the loss of DNA binding activity (Dias et al., 2003; Heine, Hernandez & Grotewold, 2004). The same phenomenon was found in a previous study, and the replacement rate and replacement location were similar (Li et al., 2016c). Furthermore, the proline (P) (located on 52) was replaced not only by S in the present study but also by alanine (A) and threonine (T) substitutions (in PeMYB30, PeMYB60 and PeMYB73). Interestingly, compared to previous studies (Du et al., 2012b; Stracke et al., 2014), more amino acid substitution sites were found in PeMYBs, which was helpful to further study on the evolutionary relationship and function of species-specific MYB TFs in moso bamboo. Considering that amino acid sequence variations may change protein function, we assumed that the species-specific PeMYBs might have new functions, which needs further research to dissect in moso bamboo.

### The expression of *PeMYB*s was tissue-specific and closely related with the development of shoots and panicles

The expression profiles of *PeMYB*s in different tissues and development stages were analyzed and described in detail, which could contribute to further study on the tissue specificity and the dynamic variation rule of *PeMYB*s in moso bamboo. The present study showed some *PeMYB*s, such as the members in S5 and S6, which showed high expression levels in all the detected tissues, suggesting that these genes might play important roles in the growth and development of bamboo by regulating the morphogenesis of various organs. In contrast, some *PeMYB*s, such as the members in S9, were expressed at low levels in all the tissues analyzed, suggesting that these genes likely have unknown functions in other tissues. This finding was consistent with that of gene structure, which further suggested that structure determines function. Genes with similar structures had similar functions and participated in the same physiological–biological processes or stages of growth and development, and their expression patterns were consistent. Interestingly, expression profiling showed that some *PeMYB*s were not detected in the young bamboo shoots, suggesting that these *PeMYB*s might mainly be involved in the synthesis of lignin and the response to environmental factors. Bamboo shoots with heights of 20 and 50 cm were in the early stages of shoot development, which was mainly in the differentiation and elongation of cells, rather than in the process of lignification.

### The function diversity and universality of PeMYBs

Although rice is phylogenetically near to moso bamboo in the phylogenetic tree (Fig. S5), most of those MYB TFs in rice have not been verified by experiment. Nevertheless, the function of *Arabidopsis* MYB TFs has been well studied and experimentally verified; thus, we predicted the function of moso bamboo MYB TFs based on the well-studied MYBs in *Arabidopsis*. The diverse gene structure implied a diverse gene functions. The MYB TFs of moso bamboo and *Arabidopsis* were divided into 36 functional clades. According to the homology of AtMYBs, the functions of different MYB members largely varied, even if these MYBs were from the same clade. For example, AtMYB4, AtMYB7 and AtMYB32 belonged to C7, and not only regulated SCW synthesis (Fornalé et al., 2010; Shen et al., 2012; Hussey et al., 2013; Yang & Wang, 2016) but also were involved in regulating anthocyanin biology and flower development (Jin et al., 2000; Preston et al., 2004; Vimolmangkang et al., 2013; Fornalé et al., 2014). The PeMYBs in C4 tended to cluster together with AtMYB16, AtMYB17 and AtMYB106, indicating that these MYBs might be related to the formation of controlling petal epidermal cell morphology and regulating early development of inflorescence (Jakoby et al., 2008; Zhang et al., 2009) and played important roles in the formation of the trichomes (Baumann et al., 2007). In addition, MYBs with the similar functions were scattered in different clades rather than aggregated in the same clade. For example, the PeMYBs involved in the formation of SCW and lignin synthesis were scattered in C1, C12, C14, C16, C22 and C32, indicating that these MYBs might play important and central roles in the SCW synthesis pathway. Importantly, nine clades did not have functional annotation due to a lack of studies in *Arabidopsis* or the species-specific clade of moso bamboo, which required further studies.

### The SCW-related *PeMYB*s showed different patterns with increasing shoot height

To further validate the reliability of the prediction, 12 *PeMYB*s related to SCW synthesis were selected to examine the changes of their transcript levels in the base region of bamboo shoots of four heights at different developmental stages by using qRT-PCR. The results showed that the transcript levels of 11 *PeMYB*s were significantly upregulated with increasing bamboo shoot height, suggesting that they might be positive regulators for the formation of SCW, which is consistent with previous studies of model plants (Zhong, Richardson & Ye, 2007; Zhong et al., 2008; Zhou et al., 2009). On the contrary, the trend of *PeMYB40* kept at a relatively stable low level after a sharp drop, indicated it might be involved in the negative regulation of SCW formation. In addition, nine *PeMYB*s demonstrated decreased expression in 6.7 m shoots compared to those in 3.0 m shoots, suggesting that these genes likely played more important roles in subsequent physiological and biochemical reactions at earlier developmental stages in bamboo shoots.

Furthermore, most members in same subgroup had similar expression patterns. Exceptionally, *PeMYB2*, *PeMYB40* and *PeMYB74* belonged to C16 showed entirely different expression patterns, suggesting that they might play different roles in the process of SCW lignification at different development stages of bamboo shoots. These results strongly supported the above functional prediction analysis for PeMYBs. However, the functional predictions performed in the present study might not be completely accurate because of the diversity of MYB TF functions between monocotyledons and dicotyledons.

Moreover, MYBs are also associated with biotic and abiotic stresses (Ashrafi-Dehkordi et al., 2018), such as powdery mildew (Li et al., 2016a), drought (Shi et al., 2018) and salt (Fang et al., 2018) and heavy metal stresses (Van De Mortel et al., 2008; Yuan et al., 2018). The regulation of PeMYBs for the growth and development of moso bamboo might involve a complex regulatory network, and more studies are needed.

## Conclusions

MYB TFs are widely distributed among higher plants and play critical roles in plant grow and development as well as in response to biotic and abiotic stresses. To reveal the status of MYBs in moso bamboo, a genome-wide screening was conducted and a total of 85 PeMYBs were identified, including 82 typical R2R3-MYB proteins, two R1R2R3-MYB proteins and one 4R-like MYB protein, which were classified into 11 subgroups (S1–S11) on the basis of their phylogenetic relationships. Analysis of intron/exon structures indicated that the splicing sites and lengths of most introns were highly conserved in the MYB domains, especially in those within the same subgroups. Based on the phylogenetic relationships and comparing with those well studied MYBs of *Arabidopsis*, the function of PeMYBs were predicted. Especially those function of 12 PeMYBs related to the biosynthesis and deposition of SCW were validated by qRT-PCR, which demonstrated their transcript abundance levels changed significantly with the increasing degree of lignification in bamboo shoots. The comprehensive analyses provided an overall insight into MYB TFs in moso bamboo and their potential involvement in SCW processes. These results will aid in understanding of and conducting further studies on the molecular mechanism of PeMYBs involved in bamboo wood formation, which is helpful for the development and utilization of bamboo lignocellulosic biomass.

## Supplemental Information

10.7717/peerj.6242/supp-1 Supplemental Information 1 List of the primersused in qRT-PCR.

10.7717/peerj.6242/supp-2 Supplemental Information 2 Gene, CDS and protein sequences of PeMYBs.

10.7717/peerj.6242/supp-3 Supplemental Information 3 Putative functions of MYB TFs in moso bamboo.

10.7717/peerj.6242/supp-4 Supplemental Information 4 Consensus sequence of R2R3-MYB domains in moso bamboo. (A) R2 Repeats; (B) R3 Repeats. The overall height of each stack indicated the conservation of the sequence at that position. The conserved tryptophan residues (Trp, W) in the MYB domain were marked with black asterisks.

10.7717/peerj.6242/supp-5 Supplemental Information 5 Multiple alignment of the amino acid sequences in R2R3-MYB domains of Arabidopsis, moso bamboo and rice.

10.7717/peerj.6242/supp-6 Supplemental Information 6 Transverse sections of vascular bundle in bamboo shoots with different heights. The blue color indicates the thickened SCW. More cells within vascular bundle were stained with increasing bamboo shoot height.
(A) 0.2 m shoots ; (B) 1. 0 m shoots ; (C) 3.0 m shoots ; (D) 6.7 m shoots. Scale bar: 200 μm.

10.7717/peerj.6242/supp-7 Supplemental Information 7 Expression analysis of selected *PeMYB*s using qRT-PCR. *PeNTB* was used as the reference gene. Average and error bars represent standard deviation of three biological replicates. Asterisks indicate a significant difference between the higher shoots and the 0.2 m shoots (* *p* < 0.05, * * *p* < 0.01).
1: 0.2 m shoots; 2: 1.0 m shoots; 3: 3.0 m shoots; 4: 6.7 m shoots.

10.7717/peerj.6242/supp-8 Supplemental Information 8 Phylogenetic tree constructed on the base of MYBs from in rice and moso bamboo. The circular unrooted tree was generated by NJ method with 1,000 bootstrap replicates.

10.7717/peerj.6242/supp-9 Supplemental Information 9 Cycle threshold (Ct) of PeMYBs and reference genes generated by qRT-PCR.

## Additional Information and Declarations

### Data Availability

The following information was supplied regarding data availability: RNA-seq data have been deposited at EMBL under accession ERP001341.

## References

[ref-1] Ashrafi-Dehkordi E, Alemzadeh A, Tanaka N, Razi H (2018). Meta-analysis of transcriptomic responses to biotic and abiotic stress in tomato. PeerJ.

[ref-2] Baumann K, Perez-Rodriguez M, Bradley D, Venail J, Bailey P, Jin HL, Koes R, Roberts K, Martin C (2007). Control of cell and petal morphogenesis by R2R3 MYB transcription factors. Development.

[ref-3] Carroll A, Somerville C (2009). Cellulosic biofuels. Annual Review of Plant Biology.

[ref-4] Cassan-Wang H, Goué N, Saidi MN, Legay S, Sivadon P, Goffner D, Grima-Pettenati J (2013). Identification of novel transcription factors regulating secondary cell wall formation in Arabidopsis. Frontiers in Plant Science.

[ref-5] Crooks GE, Hon G, Chandonia JM, Brenner SE (2004). WebLogo: a sequence logo generator. Genome Research.

[ref-6] Dias AP, Braun EL, Mcmullen MD, Grotewold E (2003). Recently duplicated maize R2R3 MYB genes provide evidence for distinct mechanisms of evolutionary divergence after duplication. Plant Physiology.

[ref-7] Du H, Feng BR, Yang SS, Huang YB, Tang YX (2012a). The R2R3-MYB transcription factor gene family in maize. PLOS ONE.

[ref-8] Du H, Wang YB, Xie Y, Liang Z, Jiang SJ, Zhang SS (2013). Genome-wide identification and evolutionary and expression analyses of MYB-related genes in land plants. DNA Research.

[ref-9] Du H, Yang SS, Liang Z, Feng BR, Liu L, Huang YB, Tang YX (2012b). Genome-wide analysis of the MYB transcription factor superfamily in soybean. BMC Plant Biology.

[ref-10] Dubos C, Stracke R, Grotewold E, Weisshaar B, Martin C, Lepiniec L (2010). MYB transcription factors in Arabidopsis. Trends in Plant Science.

[ref-11] Fan CJ, Ma JM, Guo QR, Li XT, Wang H, Lu MZ (2013). Selection of reference genes for quantitative real-time PCR in bamboo (*Phyllostachys edulis*). PLOS ONE.

[ref-12] Fang Q, Wang Q, Mao H, Xu J, Wang Y, Hu H, He S, Tu JC, Cheng C, Tian GZ, Wang XQ, Liu XP, Zhang C, Luo KM (2018). AtDIV2, an R-R-type MYB transcription factor of Arabidopsis, negatively regulates salt stress by modulating ABA signaling. Plant Cell Reports.

[ref-13] Fornalé S, Lopez E, Salazar-Henao JE, Fernández-Nohales P, Rigau J, Caparros-Ruiz D (2014). AtMYB7, a new player in the regulation of UV-sunscreens in *Arabidopsis thaliana*. Plant and Cell Physiology.

[ref-14] Fornalé S, Shi X, Chai C, Encina A, Irar S, Capellades M, Fuguet E, Torres JL, Rovira P, Puigdomènech P, Rigau J, Grotewold E, Gray J, Caparrós-Ruiz D (2010). ZmMYB31 directly represses maize lignin genes and redirects the phenylpropanoid metabolic flux. Plant Journal.

[ref-15] Gao L, Wang Z, Lin T, Li Y (2010). A comparative study of main physical and mechanical properties of *Arundinaria alpin*e and *Phyllostachys pubescens*. World Bamboo and Rattan.

[ref-16] Guo HY, Wang YC, Wang LQ, Hu P, Wang YM, Jia YY, Zhang CR, Zhang Y, Zhang YM, Wang C, Yang CP (2017). Expression of the MYB transcription factor gene *BplMYB46* affects abiotic stress tolerance and secondary cell wall deposition in *Betula platyphylla*. Plant Biotechnology Journal.

[ref-17] Guo AY, Zhu QH, Chen X, Luo JC (2007). GSDS: a gene structure display server. Hereditas.

[ref-18] He Q, Jones DC, Wei L, Xie F, Ma J, Sun R (2016). Genome-wide identification of R2R3-MYB genes and expression analyses during abiotic stress in *Gossypium raimondii*. Scientific Reports.

[ref-19] Heine GF, Hernandez JM, Grotewold E (2004). Two cysteines in plant R2R3 MYB domains participate in redox-dependent DNA binding. Journal of Biological Chemistry.

[ref-20] Hirano K, Kondo M, Aya K, Miyao A, Sato Y, Antonio BA, Namiki N, Nagamura Y, Matsuoka M (2013). Identification of transcription factors involved in rice secondary cell wall formation. Plant Cell Physiology.

[ref-21] Huang Z, Jin SH, Guo HD, Zhong XJ, He J, Li X, Jiang MY, Yu XF, Long H, Ma MD, Chen QB (2016). Genome-wide identification and characterization of TIFY family genes in moso bamboo (*Phyllostachys edulis*) and expression profiling analysis under dehydration and cold stresses. PeerJ.

[ref-22] Hussey SG, Mizrachi E, Creux NM, Myburg AA (2013). Navigating the transcriptional roadmap regulating plant secondary cell wall deposition. Frontiers in Plant Science.

[ref-23] Hussey SG, Mizrachi E, Spokevicius AV, Bossinger G, Berger DK, Myburg AA (2011). SND2, a NAC transcription actor gene, regulates genes involved in secondary cell wall development in Arabidopsis fibres and increases fibre cell area in *Eucalyptus*. BMC Plant Biology.

[ref-24] Jakoby MJ, Falkenhan D, Mader MT, Brininstool G, Wischnitzki E, Platz N, Hudson A, Hülskamp M, Larkin J, Schnittger A (2008). Transcriptional profiling of mature Arabidopsis trichomes reveals that *NOECK* encodes the MIXTA-like transcriptional regulator MYB106. Plant Physiology.

[ref-25] Jiang CZ, Gu X, Peterson T (2004). Identification of conserved gene structures and carboxy-terminal motifs in the Myb gene family of Arabidopsis and *Oryza sativa*. L. ssp. *indica*. Genome Biology.

[ref-26] Jin HL, Cominelli E, Bailey P, Parr A, Mehrtens F, Jones J, Tonelli C, Weisshaar B, Martin C (2000). Transcriptional repression by AtMYB4 controls production of UV-protecting sunscreens in Arabidopsis. EMBO Journal.

[ref-27] Jin H, Martin C (1999). Multifunctionality and diversity within the plant MYB-gene family. Plant Molecular Biology.

[ref-28] Kranz H, Scholz K, Weisshaar B (2000). c-MYB oncogene-like genes encoding three MYB repeats occur in all major plant lineages. Plant Journal.

[ref-30] Li ZJ, Peng RH, Tian YS, Han HJ, Xu J, Yao QH (2016c). Genome-wide identification and analysis of the MYB transcription factor superfamily in *Solanum lycopersicum*. Plant Cell Physiology.

[ref-31] Li YP, Tian SL, Yang XJ, Wang X, Guo YH, Ni HW (2016a). Transcriptomic analysis reveals distinct resistant response by physcion and chrysophanol against cucumber powdery mildew. PeerJ.

[ref-32] Li XL, Xue C, Li JM, Qiao X, Li LT, Yu LA, Huang YH, Wu J (2016b). Genome-wide identification, evolution and functional divergence of MYB transcription factors in Chinese white pear (*Pyrus bretschneideri*). Plant Cell Physiology.

[ref-33] Lipsick JS (1996). One billion years of MYB. Oncogene.

[ref-34] Liu CW, Fukumoto T, Matsumoto T, Gena P, Frascaria D, Kaneko T, Katsuhara M, Zhong SH, Sun XL, Zhu YM, Iwasaki I, Ding XD, Calamita G, Kitagawa Y (2013). Aquaporin OsPIP1;1 promotes rice salt resistance and seed germination. Plant Physiology and Biochemistry.

[ref-35] Liu CY, Xie T, Chen CJ, Luan AP, Long JM, Li CH, Ding YQ, He YH (2017). Genome-wide organization and expression profiling of the R2R3-MYB transcription factor family in pineapple (*Ananas comosus*). BMC Genomics.

[ref-36] McCarthy RL, Zhong R, Ye ZH (2009). MYB83 is a direct target of SND1 and acts redundantly with MYB46 in the regulation of secondary cell wall biosynthesis in Arabidopsis. Plant Cell Physiology.

[ref-37] Nakano Y, Yamaguchi M, Endo H, Rejab NA, Ohtani M (2015). NAC-MYB-based transcriptional regulation of secondary cell wall biosynthesis in land plants. Frontiers in Plant Science.

[ref-38] Niu YL, Jiang XM, Xu XY (2016). Research advances on transcription factor MYB gene family in plant. Molecular Plant Breeding.

[ref-39] Oh S, Park S, Han KH (2003). Transcriptional regulation of secondary growth in arabidopsis thaliana. Journal of Experimental Botany.

[ref-40] Pauly M, Keegstra K (2010). Plant cell wall polymers as precursors for biofuels. Current Opinion in Plant Biology.

[ref-41] Peng ZH, Lu Y, Li LB, Zhao Q, Feng Q, Gao ZM, Lu HY, Hu T, Yao N, Liu KY, Li Y, Fan DL, Guo YL, Li WJ, Lu YQ, Weng QJ, Zhou CC, Zhang L, Huang T, Zhao Y, Zhu CR, Liu XE, Yang XW, Wang T, Miao K, Zhuang CY, Cao XL, Tang WL, Liu GS, Liu YL, Chen J, Liu ZJ, Yuan LC, Liu ZH, Huang XH, Lu TT, Fei BH, Ning ZM, Han B, Jiang ZH (2013a). The draft genome of the fast-growing non-timber forest species moso bamboo (*Phyllostachys heterocycla*). Nature Genetics.

[ref-42] Peng ZH, Zhang CL, Zhang Y, Hu T, Mu SH, Li XP, Gao J (2013b). Transcriptome sequencing and analysis of the fast growing shoots of moso bamboo (*Phyllostachys edulis*). PLOS ONE.

[ref-43] Preston J, Wheeler J, Heazelwood J, Li SF, Parish RW (2004). AtMYB32 is required for normal pollen development in *Arabidopsis thaliana*. Plant Journal.

[ref-44] Salih H, Gong W, He S, Sun G, Sun JL, Du M (2016). Genome-wide characterization and expression analysis of MYB transcription factors in *Gossypium hirsutum*. BMC Genetics.

[ref-45] Scully ED, Gries T, Sarath G, Palmer NA, Baird L, Serapiglia M, Dien BS, Boateng AA, Ge Z, Funnell-Harris DL, Twigg P, Clemente TE, Sattler SE (2016). Overexpression of *SbMYB60* impacts phenylpropanoid biosynthesis and alters secondary cell wall composition in *Sorghum bicolor*. Plant Journal.

[ref-46] Seth MK (2003). Trees and their economic importance. Botanical Review.

[ref-47] Shen H, He X, Poovaiah CR, Wuddineh WA, Ma J, Mann DG, Wang H, Jackson L, Tang Y, Stewart CN JR, Chen F, Dixon RA (2012). Functional characterization of the switchgrass (*Panicum virgatum*) R2R3-MYB transcription factor PvMYB4 for improvement of lignocellulosic feedstocks. New Phytologist.

[ref-48] Shi WP, Cheng JY, Wen XJ, Wang JX, Shi GY, Yao JY, Hou LY, Sun Q, Xiang P, Yuan XY, Dong SQ, Guo PY, Guo J (2018). Transcriptomic studies reveal a key metabolic pathway contributing to a well-maintained photosynthetic system under drought stress in foxtail millet (*Setaria italica* L.). PeerJ.

[ref-49] Soler M, Camargo EL, Carocha V, Cassan-Wang H, San Clemente H, Savelli B, Hefer CA, Paiva JA, Myburg AA, Grima-Pettenati J (2015). The *Eucalyptus grandis* R2R3-MYB transcription factor family: evidence for woody growth related evolution and function. New Phytologist.

[ref-50] Stracke R, Holtgräwe D, Schneider J, Pucker B, Thomas R, Bernd W (2014). Genome-wide identification and characterisation of R2R3-MYB genes in sugar beet (*Beta vulgaris*). BMC Plant Biology.

[ref-51] Stracke R, Ishihara H, Huep G, Barsch A, Mehrtens F, Niehaus K, Weisshaar B (2007). Differential regulation of closely related R2R3-MYB transcription factors controls flavonol accumulation in different parts of the *Arabidopsis thaliana* seedling. Plant Journal.

[ref-52] Stracke R, Werber M, Weisshaar B (2001). The R2R3-MYB gene family in *Arabidopsis thaliana*. Current Opinion in Plant Biology.

[ref-53] Sun HY, Li LC, Lou YF, Zhao HS, Gao ZM (2016). Genome-wide identification and characterization of aquaporin gene family in moso bamboo (*Phyllostachys edulis*). Molecular Biology Reports.

[ref-54] Sun HY, Lou YF, Li LC, Zhao HS, Gao ZM (2017). Research advances in the growth and development of Bamboo Culm. World Forestry Research.

[ref-55] Tamura K, Stecher G, Peterson D, Filipski A, Kumar S (2013). MEGA6: molecular evolutionary genetics analysis version 6.0. Molecular Biology and Evolution.

[ref-56] Van De Mortel JE, Schat H, Moerland PD, Ver Loren Van Themaat E, Van Der EntS, Blankestijn H, Ghandilyan A, Tsiatsiani S, Aarts MG (2008). Expression differences for genes involved in lignin, glutathione and sulphate metabolism in response to cadmium in *Arabidopsis thaliana* and the related Zn/Cd-hyperaccumulator *Thlaspi caerulescens*. Plant Cell and Environment.

[ref-57] Vimolmangkang S, Han Y, Wei G, Korban SS (2013). An apple MYB transcription factor, MdMYB3, is involved in regulation of anthocyanin biosynthesis and flower development. BMC Plant Biology.

[ref-58] Wei Q, Jiao C, Guo L, Ding YL, Cao JJ, Feng JY, Dong XB, Mao LY, Sun HH, Yu F, Yang GY, Shi PJ, Ren GD, Fei ZJ (2017). Exploring key cellular processes and candidate genes regulating the primary thickening growth of moso underground shoots. New Phytologist.

[ref-59] Wilkins O, Nahal H, Foong J, Provart NJ, Campbell MM (2009). Expansion and diversification of the *Populus* R2R3-MYB family of transcription factors. Plant Physiology.

[ref-60] Windham GL, Williams WP, Mylroie JE, Reid CX, Womack ED (2018). A histological study of aspergillus flavus colonization of wound inoculated maize kernels of resistant and susceptible maize hybrids in the field. Frontiers in Microbiology.

[ref-61] Wu HL, Lv H, Li L, Liu J, Mu SH, Li XP, Gao J (2015). Genome-wide analysis of the AP2/ERF transcription factors family and the expression patterns of DREB genes in Moso Bamboo (*Phyllostachys edulis*). PLOS ONE.

[ref-62] Yang JH, Wang HZ (2016). Molecular mechanisms for vascular development and secondary cell wall formation. Frontiers in Plant Science.

[ref-63] Yu HQ (2003). Study on property of bamboo culms. World Bamboo and Rattan.

[ref-64] Yuan JB, Bai YQ, Chao YH, Sun XB, He CY, Liang XH, Xie LJ, Han LB (2018). Genome-wide analysis reveals four key transcription factors associated with cadmium stress in creeping bentgrass (*Agrostis stolonifera* L.). PeerJ.

[ref-65] Zhang Y, Cao G, Qu LJ, Gu H (2009). Characterization of Arabidopsis MYB transcription factor gene AtMYB17 and its possible regulation by LEAFY and AGL15. Journal of Genetics and Genomics.

[ref-66] Zhang YX, Han XJ, Sang J, He XL, Liu MY, Qiao GR, Zhuo RY, He GP, Hu JJ (2016). Transcriptome analysis of immature xylem in the Chinese fir at different developmental phases. PeerJ.

[ref-68] Zhao HS, Peng ZH, Fei BH, Li LB, Hu T, Gao ZM, Jiang ZH (2014). BambooGDB: a bamboo genome database with functional annotation and an analysis platform. Database.

[ref-69] Zhong R, Lee C, Zhou J, Mccarthy R, Ye ZH (2008). A battery of transcription factors involved in the regulation of secondary cell wall biosynthesis in Arabidopsis. Plant Cell.

[ref-70] Zhong R, Richardson EA, Ye ZH (2007). The MYB46 transcription factor is a direct target of SND1 and regulates secondary wall biosynthesis in Arabidopsis. Plant Cell.

[ref-71] Zhou J, Lee C, Zhong R, Ye ZH (2009). MYB58 and MYB63 are transcriptional activators of the lignin biosynthetic pathway during secondary cell wall formation in Arabidopsis. Plant Cell.

