# Supplementary material for: Genome-wide identification and expression analysis of the MYB transcription factor in moso bamboo (Phyllostachys edulis)
Source: PeerJ. 2019 Jan 11;6:e6242. doi: 10.7717/peerj.6242 (PMC6331034; doi:10.7717/peerj.6242)
Supplement: Supplemental Information 1 [file peerj-07-6242-s001.doc]

| **Gene name** | **Primer sequence** |
| --- | --- |
| *PeMYB3* | F: 5′-GCAGTGTGCTCTATGGTGTGC-3′  R: 5′-ATGTGCTGTTGGTGACCTGC-3′ |
| *PeMYB10* | F: 5′-ACGGCAACGCGACAGAGG-3′  R: 5′-CACGCCTGGCTTTGTGGG-3′ |
| *PeMYB14* | F: 5′-CGGAGTCGAGCACGAACA-3′  R: 5′-GCGATGCTGCTGAACTCG-3′ |
| *PeMYB22* | F: 5′-GCAGCGGCAATGGTTCA-3′  R: 5′-TGCCAATGCCAGTGCCT-3′ |
| *PeMYB26* | F: 5′-TGATTGGGACCAGGGCAAA-3′  R: 5′-CCTCCCTCTTCACAGGCTTCAT-3′ |
| *PeMYB29* | F: 5′-CCACAAGGTCAGCCGGGT-3′  F: 5′-GATCCATCTGGTTACTTGTGAGAGC-3′ |
| *PeMYB33* | F: 5′-AATCTAGCGACACGATAAATCG-3′  R: 5′-CCGCCAGTGTTCCTGCT-3′ |
| *PeMYB37* | F: 5′-CCATGCCTGCTACGAGCAT-3′  R: 5′-TGGTGTTAGTTCTTGATCCCACAT-3′ |
| *PeMYB40* | F: 5′-CGGCGATGGTGCCCATAT-3′  R: 5′-TGACGTCGAGAGGGCTGATG-3′ |
| *PeMYB50* | F: 5′-GACTTTCTCCAAGTGAATGTGAGCT-3′  R: 5′-TCTATGAGCTGCACCTGCCCT-3′ |
| *PeMYB64* | F: 5′-ACGAGCAAGGAGCCTGACA-3′  R: 5′-CGATGATGCTCCAAATGTCC-3′ |
| *PeMYB74* | F: 5′-CAGCAGGGATAGTGGAGGCA-3′  R: 5′-TTGAGCCCTGTTGGTGTTGAG-3′ |
| *PeNTB* | F: 5′-TCTTGTTTGACACCGAAGAGGAG-3′  R: 5′-AATAGCTGTCCCTGGAGGAGTTT-3′ |
| *PeTIP41* | F: 5′-AAAATCATTGTAGGCCATTGTCG-3′  R: 5′-ACTAAATTAAGCCAGCGGGAGTG-3′ |
