# Supplementary material for: Genome-wide identification and expression analysis of the MYB transcription factor in moso bamboo (Phyllostachys edulis)
Source: PeerJ. 2019 Jan 11;6:e6242. doi: 10.7717/peerj.6242 (PMC6331034; doi:10.7717/peerj.6242)
Supplement: Supplemental Information 3 [file peerj-07-6242-s003.doc]

| **Subclades** | **Moso bamboo** | **Arabidopsis** | **Rice** | **Functions** |
| --- | --- | --- | --- | --- |
| C1 (S3) | PeMYB10  PeMYB29  PeMYB64 | AtMYB10  AtMYB72  AtMYB58 AtMYB63 | OsMYB56  OsMYB30 | Lignin biosynthesis  Respond to conditions of iron deficiency |
| C2 (S2) | PeMYB45  PeMYB53  PeMYB57 | AtMYB13  AtMYB14 AtMYB15 |  | Involved in cold stress tolerance |
| C3 | PeMYB25 |  |  | Unknown |
| C4 (S9) | PeMYB31 | AtMYB17 |  | A repressor of cell outgrowth  Epidermal cells |
|  | PeMYB79  PeMYB11  PeMYB65  PeMYB23  PeMYB36 | AtMYB106  AtMYB16 |  |
| C5 (S14) |  | AtMYB84  AtMYB68  AtMYB87  AtMYB36  AtMYB38  AtMYB37 |  | Vegetative development |
| C6 (S1) | PeMYB2  PeMYB13  PeMYB70  PeMYB43  PeMYB4  PeMYB19  PeMYB15  PeMYB18  PeMYB77  PeMYB82 | AtMYB60  AtMYB94  AtMYB96  AtMYB31  AtMYB30 |  | Respond to environmental stress by regulating hypersensitive cell death responses  Activate cuticular wax biosynthesis  Respond to environmental stress by regulating ABA-dependent signaling pathways |
| C7 (S4) | PeMYB26 | AtMYB7  AtMYB32  AtMYB4 | OsMYB97  OsMYB91 | Anthocyanin biosynthesis and flower development; transcriptional silencing  Negative regulators for the NAC domain master regulators |
| C8 | PeMYB76  PeMYB16  PeMYB59 |  |  | Unknown |
| C9 (S12) |  | AtMYB51  AtMYB122  AtMYB34  AtMYB28  AtMYB29  AtMYB76 |  | Glucosinolate biosynthesis |
| C10 (S24) |  | AtMYB92  AtMYB53  AtMYB92 |  | Root development |
| C11 (S10) |  | AtMYB39  AtMYB107  AtMYB9 |  | Trichome development |
| C12 (S8) | PeMYB33  PeMYB55  PeMYB14  PeMYB5  PeMYB81  PeMYB44  PeMYB39  PeMYB61  PeMYB54  PeMYB66 | AtMYB42  AtMYB85  AtMYB20  AtMYB43 | OsMYB96  OsMYB85  OsMYB93  OsMYB32  OsMYB69 | Lignin biosynthesis |
| C13 (S11) | PeMYB20 | AtMYB49  AtMYB41  AtMYB74  AtMYB102 |  | Respond to biotic stress  Resistance to salt  Resistant to drought |
| C14 (S26) | PeMYB35  PeMYB37 | AtMYB103  AtMYB67  AtMYB26 | OsMYB83  OsMYB13  OsMYB77 | Secondary wall deposition in anthers |
| C15 | PeMYB71  PeMYB80  PeMYB17  PeMYB68 |  |  | Unknown |
| C16 (S13) | PeMYB22  PeMYB40  PeMYB69  PeMYB74 | AtMYB86  AtMYB55  AtMYB61  AtMYB50 | OsMYB58  OsMYB5 | Lignin deposition and stomatal aperture |
| C17 (S7) | PeMYB30  PeMYB60  PeMYB73 | AtMYB12  AtMYB11  AtMYB111 |  | Flavonol biosynthesis |
| C18 (S15) |  | AtMYB0  AtMYB66  AtMYB23 |  | Epidermal cells |
| C19 | PeMYB7  PeMYB47 |  |  | Unknown |
| C20 (S5) | PeMYB72 | AtMYB123 |  | Biosynthesis of proanthocyanidins (PAs) |
| C21 (S6) |  | AtMYB114  AtMYB113  AtMYB75  AtMYB90 |  | Anthocyanin biosynthesis |
| C22 (S27) | PeMYB3 | AtMYB46  AtMYB83 |  | Lignin biosynthesis |
| C23 (S16) |  | AtMYB18  AtMYB19  AtMYB45 |  | Response to far-red light |
| C24 | PeMYB32  PeMYB1  PeMYB8 |  |  | Unknown |
| C25 (S17) | PeMYB27 | AtMYB71  AtMYB79  AtMYB121 |  | Root development |
| C26 | PeMYB52 |  |  | Unknown |
| C27 (S19) |  | AtMYB21  AtMYB24 |  | Anther development |
| C28 (S20) | PeMYB34  PeMYB24  PeMYB42 PeMYB38  PeMYB75 | AtMYB116  AtMYB62  AtMYB108 AtMYB78  AtMYB112  AtMYB2 |  | Stress responses |
| C25 (S17) | PeMYB58  PeMYB41 PeMYB48  PeMYB63 | AtMYB48  AtMYB59 |  | Root development |
| C29 (S18) | PeMYB6 PeMYB9 | AtMYB104  AtMYB81 AtMYB97  AtMYB120 AtMYB101  AtMYB65 AtMYB33 |  | Involved in anther/pollen development |
| C30 | PeMYB28 PeMYB3R-1  PeMYB67  PeMYB3R-2 | AtMYB3R-3 AtMYB3R-5  AtMYB3R-2 AtMYB3R-1  AtMYB3R-4 |  | Unknown |
| C31 (S25) | PeMYB51 | AtMYB100  AtMYB22 AtMYB118  AtMYB115 AtMYB98 AtMYB64 AtMYB119 |  | Embryogenesis  Synthesis of polyunsaturated (PUFA) |
| C32 (S21) | PeMYB62  PeMYB50  PeMYB21 | AtMYB117  AtMYB105 AtMYB69  AtMYB110 AtMYB56 AtMYB52  AtMYB54 | OsMYB101  OsMYB45  OsMYB87  OsMYB4 | Lignin, xylan and cellulose biosynthesis |
| C33 (S23) |  | AtMYB25 AtMYB109  AtMYB1 |  | Abiotic stresses |
| C34 (S22) | PeMYB12 PeMYB78  PeMYB49  PeMYB56 | AtMYB70 AtMYB73 AtMYB77  AtMYB44 |  | Abiotic stresses |
| C35 | PeMYB4R-1 | AtMYB4R-1 |  | Unknown |
| C36 | PeMYB46 |  |  | Unknown |
