# Supplementary figures and images for: Genome-wide identification and expression analysis of the MYB transcription factor in moso bamboo (Phyllostachys edulis)
Source: PeerJ. 2019 Jan 11;6:e6242. doi: 10.7717/peerj.6242 (PMC6331034; doi:10.7717/peerj.6242)

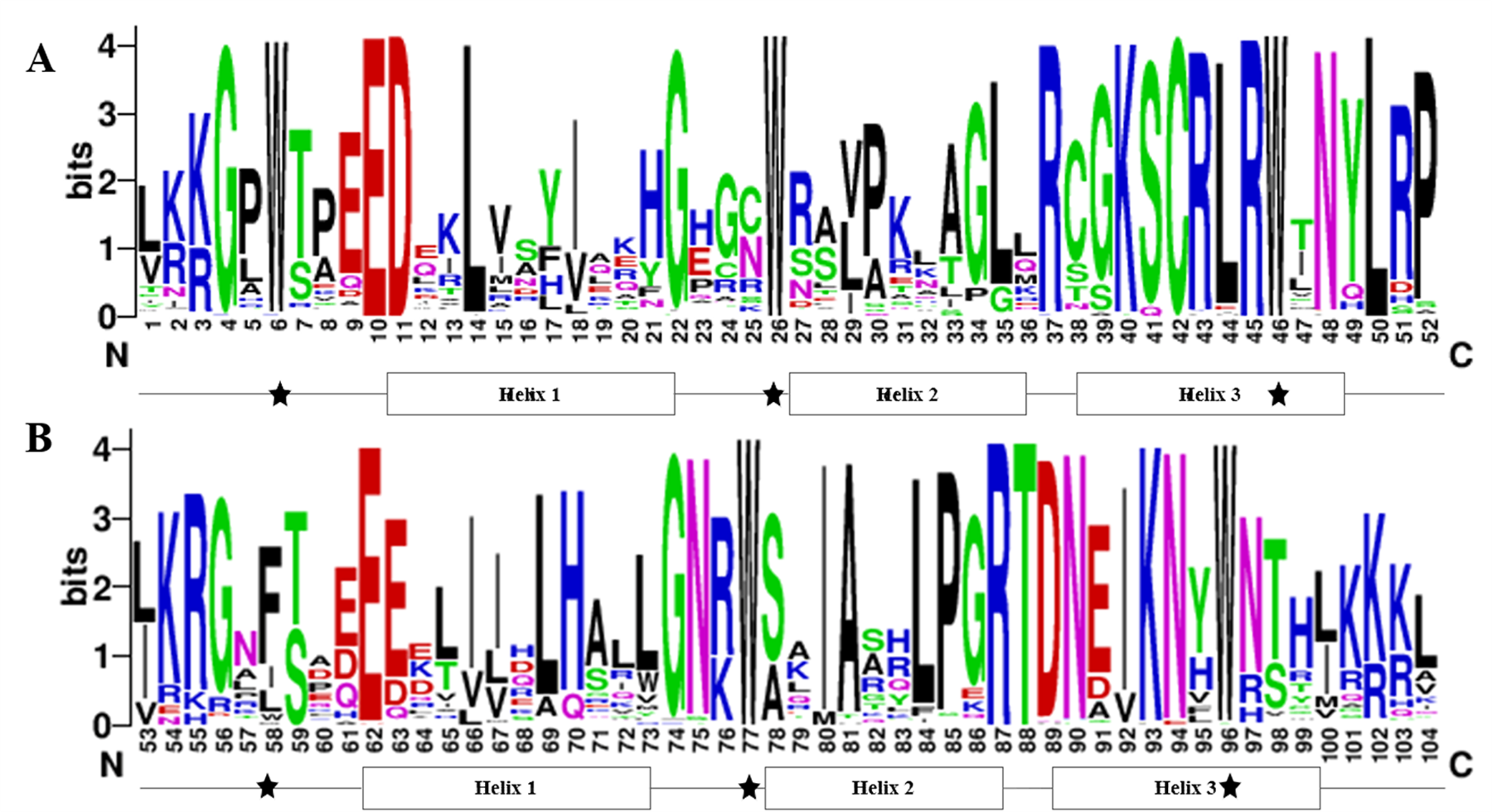

Supplement: Supplemental Information 4 — (A) R2 Repeats; (B) R3 Repeats. The overall height of each stack indicated the conservation of the sequence at that position. The conserved tryptophan residues (Trp, W) in the MYB domain were marked with black asterisks. [file peerj-07-6242-s004.png]

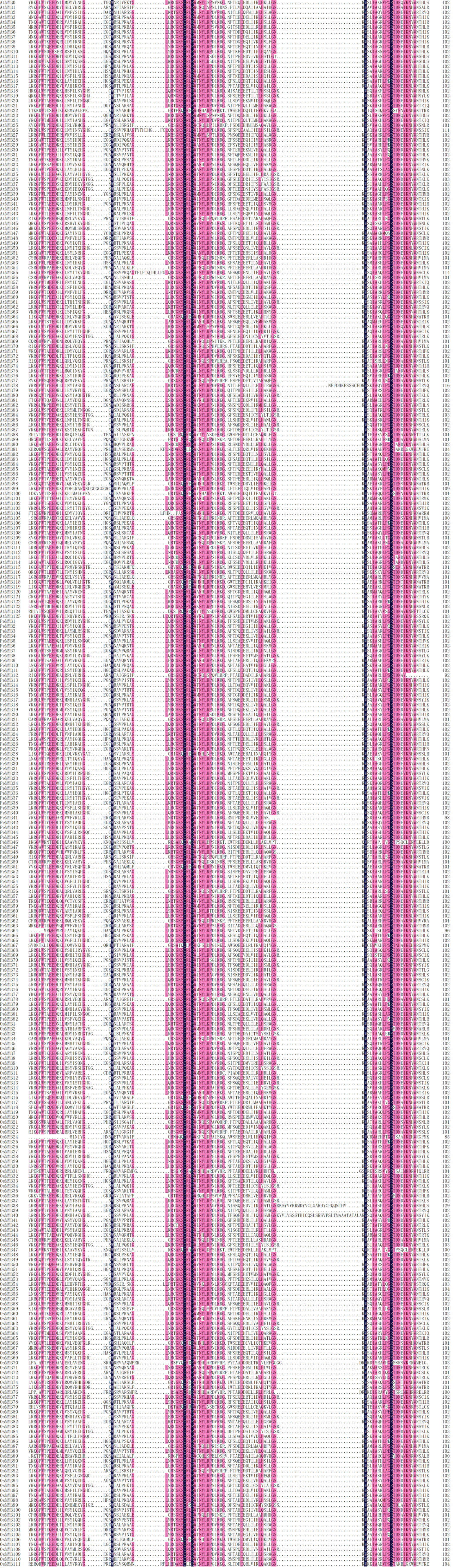

Supplement: Supplemental Information 5 [file peerj-07-6242-s005.png]

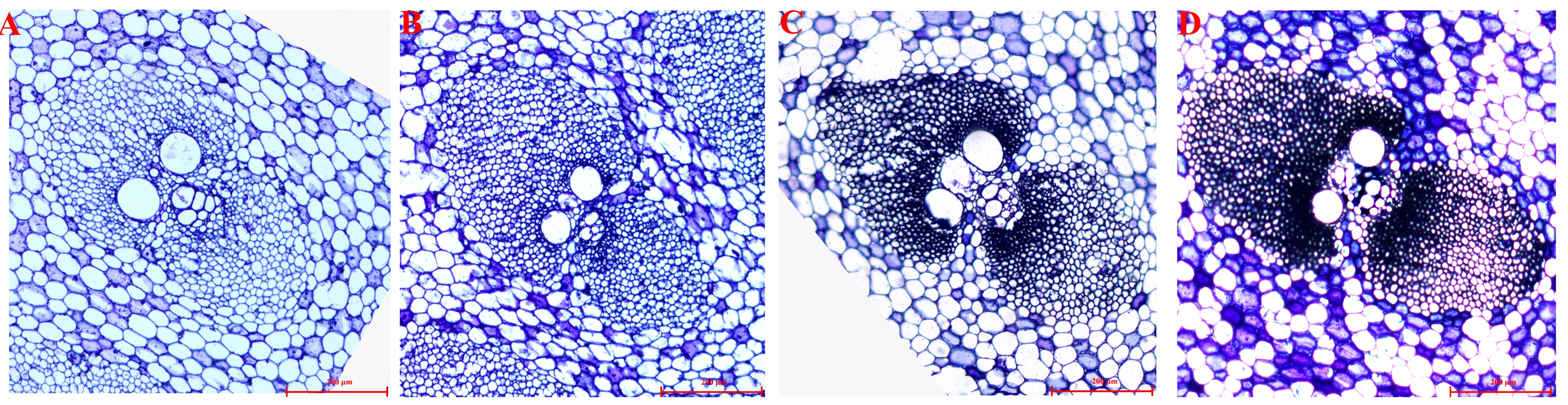

Supplement: Supplemental Information 6 — The blue color indicates the thickened SCW. More cells within vascular bundle were stained with increasing bamboo shoot height. (A) 0.2 m shoots ; (B) 1. 0 m shoots ; (C) 3.0 m shoots ; (D) 6.7 m shoots. Scale bar: 200 μm. [file peerj-07-6242-s006.png]

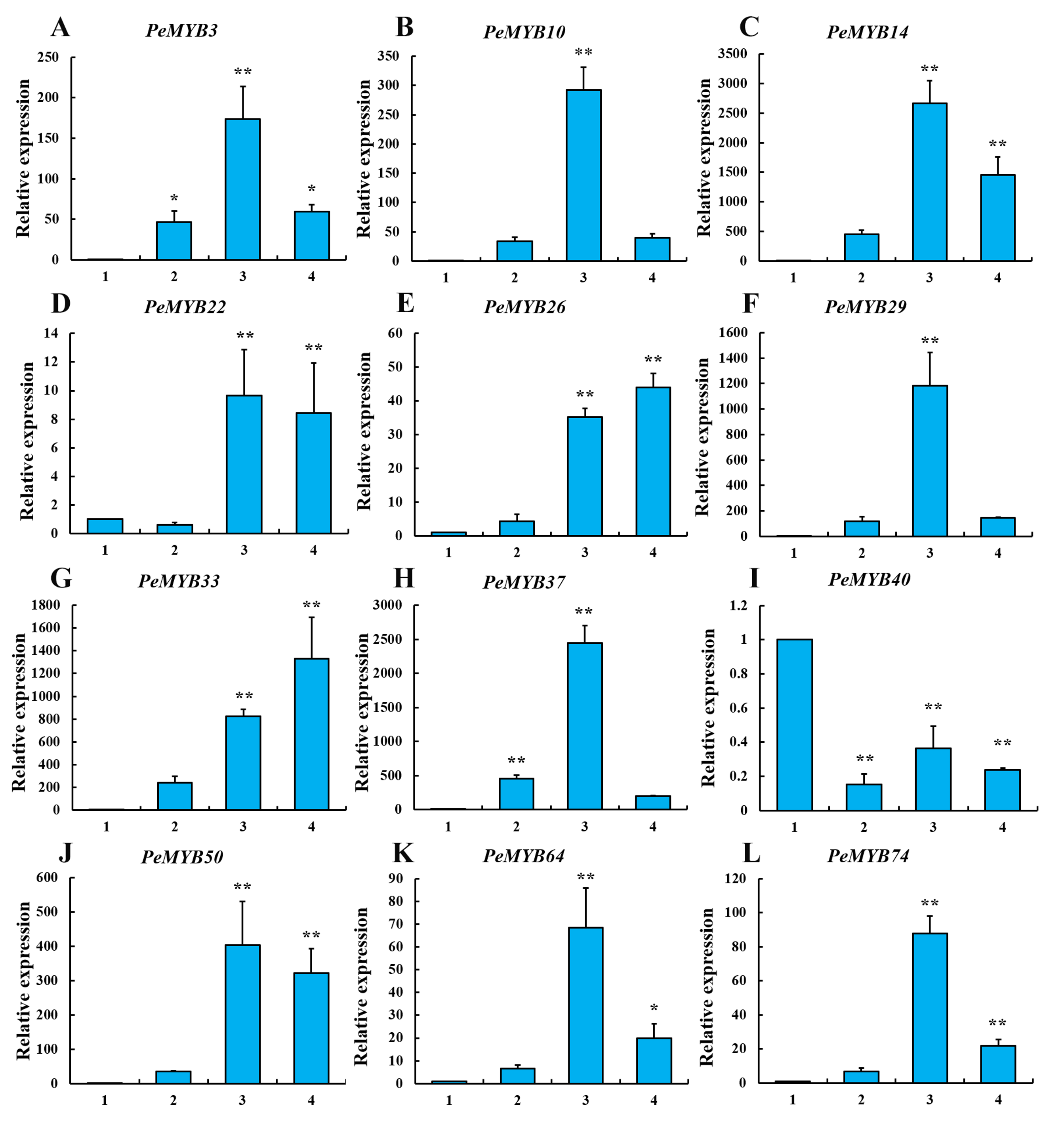

Supplement: Supplemental Information 7 — PeNTB was used as the reference gene. Average and error bars represent standard deviation of three biological replicates. Asterisks indicate a significant difference between the higher shoots and the 0.2 m shoots (* p < 0.05, * * p < 0.01). 1: 0.2 m shoots; 2: 1.0 m shoots; 3: 3.0 m shoots; 4: 6.7 m shoots. [file peerj-07-6242-s007.png]

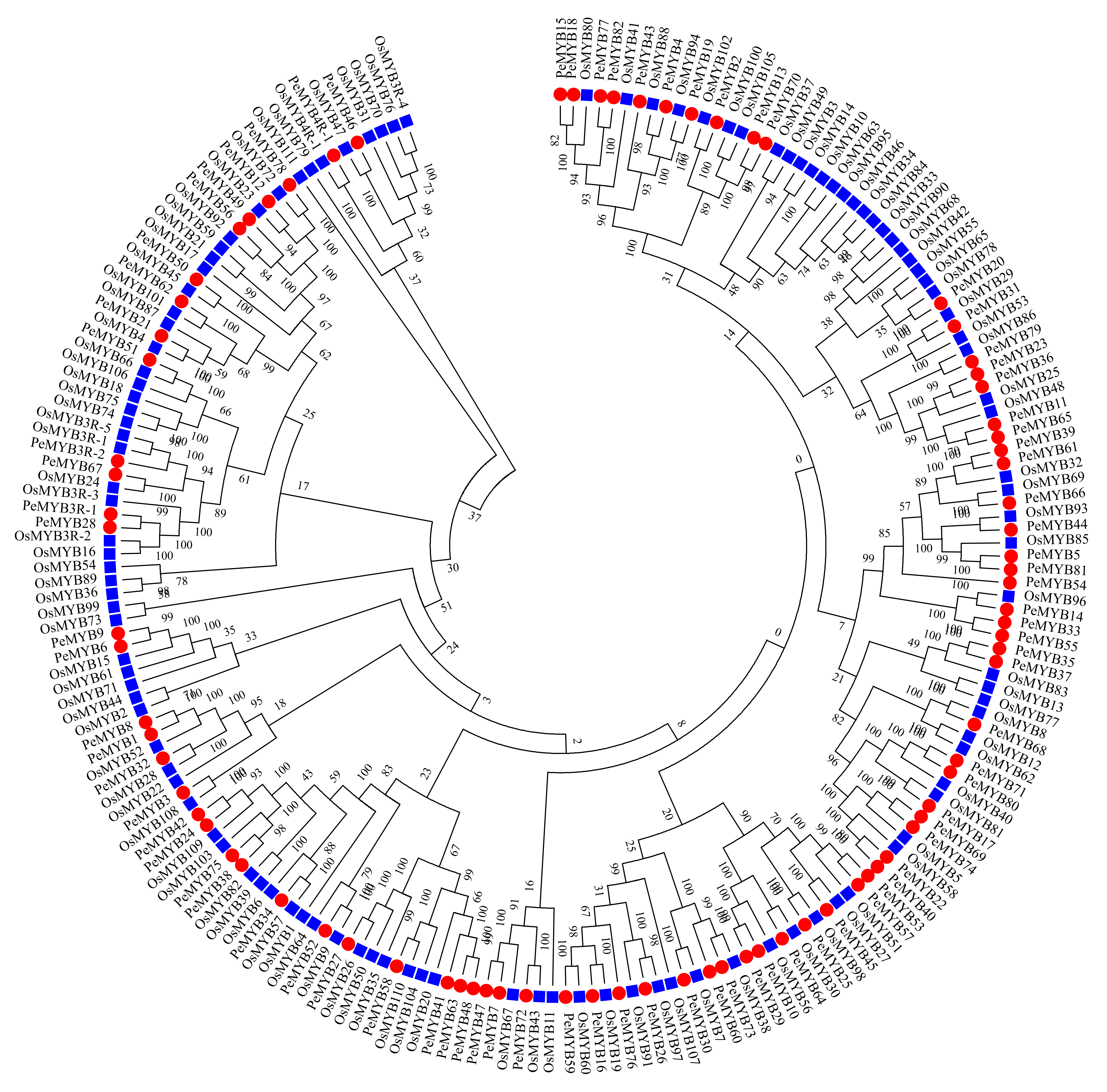

Supplement: Supplemental Information 8 — The circular unrooted tree was generated by NJ method with 1,000 bootstrap replicates. [file peerj-07-6242-s008.png]
